# Identification of tumor-promoting functions of the Homeobox family transcription factor MSX1 in cervical cancer

**DOI:** 10.1038/s41420-026-03191-y

**Published:** 2026-06-05

**Authors:** Paulina Brücker, Svenja Horn, Shaishavi Jansari, Marianna Kombou, Simon Eppich, Udo Jeschke, Samy Hakroush, Stefan Küffer, Sonja Fritzsche, Florian Wegwitz, Julia Gallwas

**Affiliations:** 1 Department of Gynecology and Obstetrics, University Medical Center Göttingen, Göttingen, Germany; 2 Department of Obstetrics and Gynecology, University Hospital, Ludwig Maximilians University, Munich, Germany; 3 Gynecology, Faculty of Medicine, University of Augsburg, Augsburg, Germany; 4 Institute of Pathology, University Medical Center Göttingen, Göttingen, Germany

**Keywords:** Cervical cancer, Tumour biomarkers, Cancer epigenetics

## Abstract

Cervical cancer (CC) remains a leading cause of cancer-related mortality among women, particularly in underdeveloped or developing countries lacking adequate screening programs. Despite being highly preventable through HPV vaccinations and screening tests, and highly treatable when detected early, CC continues to pose a significant health burden. MSX1, a transcription factor of the homeobox family, plays an important role in the development and differentiation of tissues, including the gynecological tract. Since MSX1 has been identified as both a tumor suppressor and an oncogene in tumorigenesis, but its function in CC remains elusive to date, the present study aims to elucidate the role of MSX1 in CC and its precancerous stages. Our results demonstrate that MSX1 acts as a tumor-promoting factor in CC. While normal squamous epithelium of the cervix is negative for MSX1, we observed de novo expression of MSX1 in precancerous lesions. We found that MSX1 expression enhances the clonogenic and migratory capacities of cervical cancer cell lines. Mechanistic insights revealed an MSX1-dependent induction of the epithelial-to-mesenchymal transition (EMT), a process highly correlated with cancer development and progression. CUT&RUN sequencing data identified enriched signaling of the cytoskeletal modulators RHO/RAC/CDC42 in MSX1-expressing cells, potentially mediated by MSX1. Additionally, FOS, a downstream effector of RHO, was found as a key factor contributing to CC aggressiveness. Interference with both RHO-signaling and FOS-mediated regulation completely reversed the aggressive phenotype of MSX1-expressing cells. In conclusion, our findings suggest that MSX1 plays a crucial role in cancer-related signaling pathways and may have implications for the development of therapeutic approaches in cervical cancer.

## Introduction

Cervical cancer (CC) is the fourth most common cancer among women globally and ranks as the eighth most common cancer overall [1]. In 2022, global records indicated over 600,000 new diagnoses of CC. Despite being highly preventable and treatable when detected early, CC remains a primary cause of cancer-related deaths in women worldwide [2]. About 85% of these fatalities occur in underdeveloped or developing nations, where adequate screening is hindered by poor infrastructure and medical resources. Consequently, the mortality rate is 18 times higher in low and middle-income countries compared to wealthier nations [3]. Based on global cancer statistics (GLOBOCAN) in 2022, an estimated 94% of the 350,000 CC deaths were reported in low- and middle-income countries [1].

Human Papillomavirus (HPV) infection is a predominant cause of CC, with HPV DNA detected in ~95% of malignant cervical lesions. High-risk HPV serotypes, particularly types 16 and 18, are implicated in around 70% of cases [4]. Although HPV is a common sexually transmitted infection often cleared by the immune system, persistent cases can lead to malignant transformation [5]. The increased expression of viral oncogenes E6/E7, which inactivate cellular proteins p53 and Rb, marks the transition from mild dysplasia to invasive cancer by disrupting normal cellular regulatory pathways [6]. Consequently, preventive measures such as HPV vaccination and routine screening have contributed to significantly reduced CC risk [7]. Other factors increasing CC risk include smoking, prolonged oral contraceptive use, high parity, and co-infections with herpes simplex virus type 2 or human immunodeficiency virus [8].

The cervix, situated at the lower end of the uterus and connecting to the vaginal canal, comprises the outer ectocervix, with its stratified squamous epithelium, and the inner endocervix, lined with mucus-secreting glandular epithelium [9]. CC typically evolves over time, beginning with dysplasia, in which abnormal cellular changes occur in the cervical tissue. If untreated, these dysplastic cells can progress to cancerous cells, invading deeper cervical tissues and adjacent areas. Most CCs originate at the squamocolumnar junction, also known as the transformation zone, where these two epithelial types converge. Up to 90% of CC cases are squamous cell carcinomas, while adenocarcinomas arise from glandular cells and are less prevalent, with adenosquamous carcinoma, a rare hybrid type, also observed [2].

The treatment strategies for CC are guided by the International Federation of Gynecology and Obstetrics (FIGO) stage classification. Surgery often serves as the initial approach to eliminate malignant tissues, supplemented by platinum-based chemotherapy and radiation therapy [8]. However, a significant subset of patients does not respond adequately to these standard treatments [10]. Despite advancements in prevention, there is an urgent need to understand CC’s tumorigenesis comprehensively and identify reliable prognostic biomarkers. One promising candidate is MSX1, also known as HOX7, a transcription factor in the homeobox protein family located on chromosome 4p16.2 [11]. Mainly acting as a transcriptional repressor, MSX1 is crucial in various developmental processes [12] and has been associated with several cancers, including gynecological malignancies [13, 14]. Although genetic mutations in MSX1 are rare in cancers, transcriptional dysregulation is prevalent, often via promoter methylation, which can lead to MSX1 inactivation, suggesting a potential biomarker role [15]. MSX1 dysregulation influences crucial cellular processes like proliferation, differentiation, and apoptosis, leading to diverse cancer outcomes [16]. In certain malignancies, its aberrant activity is associated with aggressive tumor phenotypes, while in others, it exhibits tumor-suppressive functions, notably in gynecological contexts [17]. Therefore, understanding MSX1’s dual role as a tumor suppressor and promoter is vital. Evidence on MSX1’s function in CC remains limited. Notably, its expression is reduced in malignant cervical tissues compared to normal ones [15], hinting at a potential tumor-suppressive role. Thus, exploring MSX1’s involvement may pave the way for innovative therapeutic interventions. This study aims to clarify MSX1’s role in CC and its potential as a diagnostic and therapeutic target.

## Results

### MSX1 is re-expressed in pre-cancerous cervical lesions and associated with CC cell aggressiveness

**Fig. 1:**
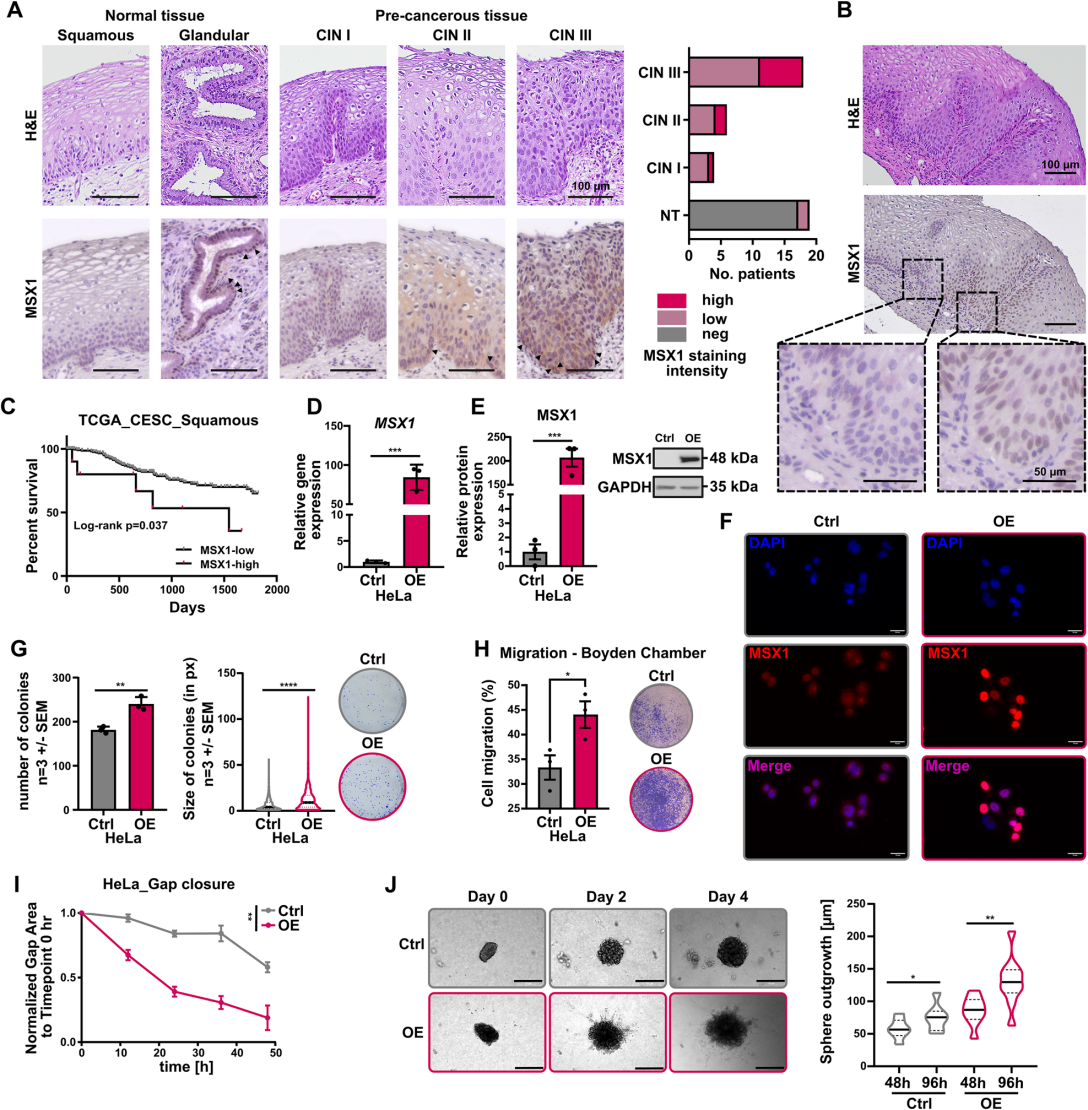
*MSX1* re-expression correlates with tumorigenic properties in CC cells. **A**, **B** Hematoxylin and Eosin (H&E) staining and MSX1 immunohistochemical (IHC) staining of normal and pre-cancerous cervical lesions (Scale bar: **A**, **B** upper panels**:** 100 µm; **B** lower panels: 50 µm). *p* < 0.0001; *n* = 47. **C** Survival analysis from the publicly available TCGA-CESC dataset showing poor prognosis in patients with high *MSX1* expression. **D**–**F** Validation of *MSX1* expression in HeLa cells with stable overexpression (OE) compared to the controls (Ctrl) via RT-qPCR (**D**), Western blot (**E**) and immunofluorescence (IF) staining (Scale bar: 10 µm) (**F**). Assessment of MSX1-mediated tumorigenic properties in HeLa Ctrl and HeLa OE cells via colony formation assay (**G**), Boyden chamber assay (**H**), gap closure assay (**I**) and sphere outgrowth measurement (**J**; Scale Bar: 10 µM). **D**–**J**: *n* = *3* biological replicates. Statistical test: **A**, **B**: Chi-Square test, **C**: Log-rank test, **D**, **E**, **G**, **H**, **J**: Student’s *t* test, **I**: AUC follow by Student’s *t* test. *p-val < 0.05, **p-val ≤ 0.01, ****p-val ≤ 0.0001.

Several reports have shown that the MSX1 transcription factor is expressed in normal epithelial tissues of gynecological organs [15, 18]. However, its precise distribution in the tissue structures of the cervix has not been well characterized yet. Interestingly, Xu and colleagues suggested that MSX1 expression might be increased in pre-cancerous tissues of the cervix compared to the normal counterparts [19]. In contrast, Yue reported a potential tumor suppressive function of MSX1 in cervical cancer [15]. To better understand MSX1 function in cervical tissues, we performed immunohistochemical (IHC) staining on normal as well as pre-cancerous cervical intraepithelial neoplasia I to III (CIN I, II and III). Staining of normal tissues revealed that squamous epithelial cells of the ectocervix do not express MSX1 whereas glandular structures of the endocervix show strong nuclear staining (Fig. 1A). This overall pattern was reproduced in the respective malignancies of the cervix with a clear tendency for high *MSX1* expression in adenocarcinoma and low *MSX1* expression in squamous cell carcinoma (SCC) at mRNA (TCGA-CESC, Supplementary Fig. S1A) and protein level (own dataset, Supplementary Fig. S1B). Surprisingly, IHC staining of pre-cancerous lesions indicated a gradual re-expression of MSX1 along the progression grade of the CIN (Fig. 1A). Neighboring normal squamous tissues remained negative, arguing for a specific *de-novo* expression in the CIN lesions (Fig. 1B). This intriguing behavior could point at an active involvement of MSX1 in the development of the malignancy. Indeed, although *MSX1* levels are overall reduced in CC tissues, and particularly in SCC, a small fraction of patients harbors high *MSX1* levels and simultaneously showed significant reduced survival compared to patients with low *MSX1* expression (Fig. 1C). To investigate this phenomenon, we established stable *MSX1* overexpression (OE) in two different MSX1 negative CC cell lines, HeLa and SiHa, using the Sleeping Beauty vector technology. The transgene overexpression was verified by real-time quantitative PCR (RT-qPCR), Western blot and immunofluorescence (Fig.1D–F and Supplementary Fig. S1C). Interestingly, *MSX1* OE did not influence the proliferation of the two cell lines (Supplementary Fig. S1D). However, their capacity to grow as single cells under adherent conditions and form colonies was significantly increased compared to the parental counterparts (Fig. 1G and Supplementary Fig. S1E). Additionally, the motility of the HeLa cells was strongly increased upon *MSX1* OE, as assessed via Boyden chamber, gap closure, and 3D extracellular matrix invasion assays (Fig. 1H–J, Supplementary Fig. S1F). Overall, our data on *MSX1* re-expression in CC cells indicate a potential stimulation of oncogenic features increasing their aggressiveness.

### MSX1 induces epithelial-mesenchymal transition (EMT) in CC cells

**Fig. 2:**
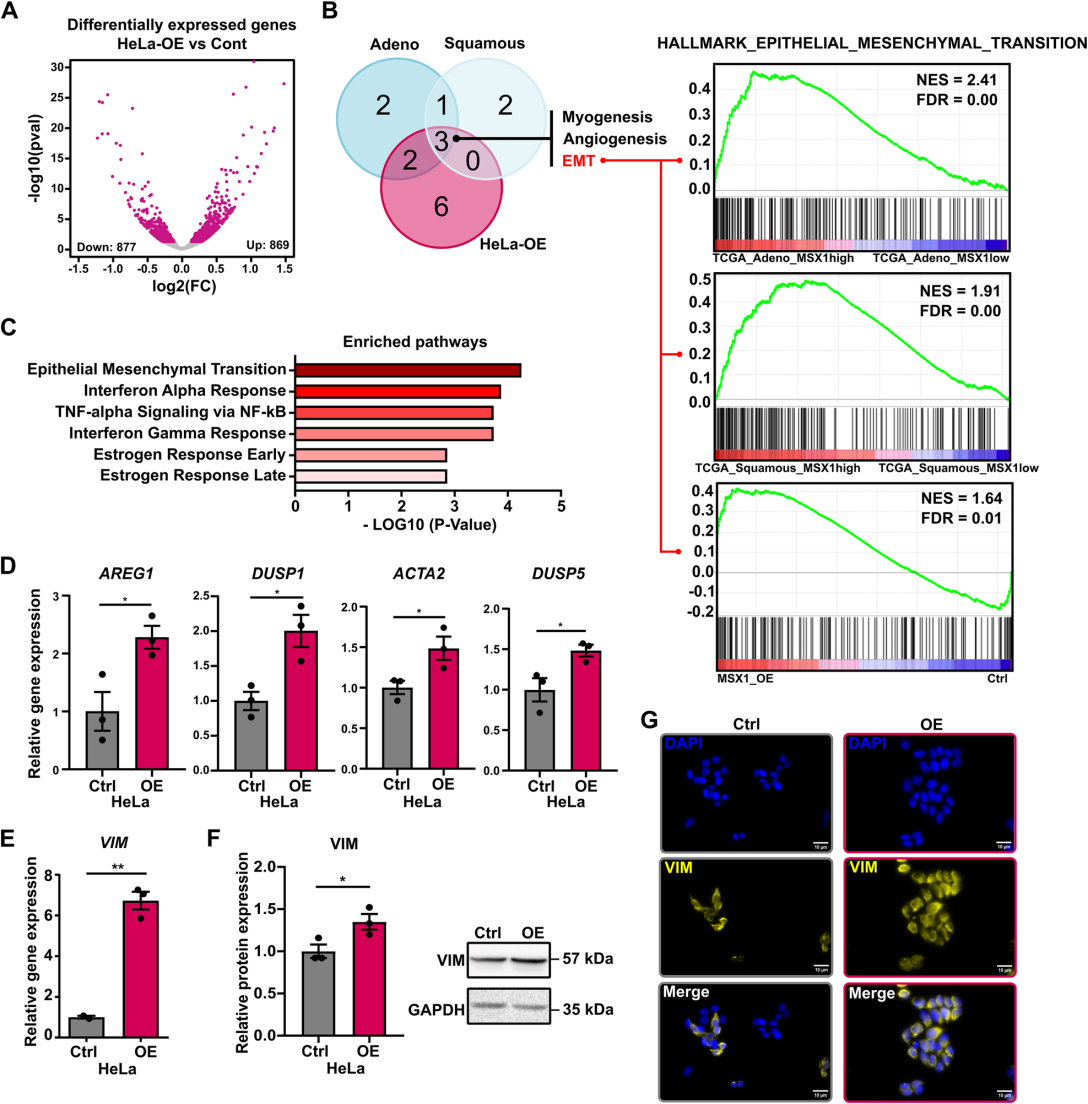
High *MSX1* expression favors EMT phenotype in CC cells. **A** mRNA-sequencing data identified 869 up- and 877 downregulated genes in HeLa cells upon *MSX1* OE (*p*val < 0.05, norm. counts > 10). **B**, **C** *MSX1*-high CC cells enrich the ‘HALLMARK_EPITHELIAL_MESENCHYMAL_TRANSITION’ gene set: GSEA plots of HeLa cells OE vs ctrl as well as patients of the TCGA_CESC dataset (squamous and adeno carcinoma patients are separately depicted). NES Normalized Enrichment Score, FDR False Discovery Rate. **D** RT-qPCR validating upregulation of selected enriched genes of the ‘HALLMARK_EPITHELIAL_MESENCHYMAL_TRANSITION’ signature upon MSX1 OE. HeLa cells upregulate vimentin at mRNA and protein levels, as assessed by RT-qPCR (**E**), Western blot (**F**) and immunofluorescence (**G**). Scale bar = 10 µm. All experiments were performed in *n* = *3* biological replicates. Statistical test: **D**–**F**: Student’s *t*-test. *p-val < 0.05, **p-val ≤ 0.01.

To better understand the molecular mechanisms underlying MSX1-mediated increased aggressiveness in CC cells, we performed mRNA sequencing in HeLa cells with or without MSX1 OE. Differential gene expression analysis using the DESeq2 tool revealed 869 and 877 significantly UP and DN regulated genes, respectively (Fig. 2A). Subsequent gene set enrichment analyses (GSEA) identified the “Epithelial Mesenchymal Transition” signature as most strongly enriched in HeLa cells overexpressing *MSX1* (Fig. 2B, C). Interestingly, the same EMT gene set was also enriched in MSX1^high^ CC patients with adeno- or squamous cell carcinoma in the TCGA-CESC dataset, supporting a possible implication of MSX1 in the induction of this signature. We confirmed the up regulation of the most strongly regulated genes of the EMT-signature in HeLa and SiHa cells upon MSX1 OE by RT-qPCR (Fig. 2D and Supplementary Fig. S2A). Strikingly, the expression of the prominent EMT marker vimentin was also strongly increased at the mRNA and protein level in MSX1 OE cells (Fig. 2E, F). Enhanced vimentin amount in MSX1 OE cells was also visualized via immunofluorescence (Fig. 2G). Finally, MSX1 knockdown (KD) in MSX1 OE cells reduced the expression of EMT-related genes, validating its direct regulatory role (Supplementary Fig. S2B, C). Together, re-expression of MSX1 in CC cell lines induces EMT states that correlate with increased aggressiveness.

### MSX1 promotes the transcription of genes of the RHO/RAC/CDC42 signaling pathway by directly binding to their regulatory regions

**Fig. 3:**
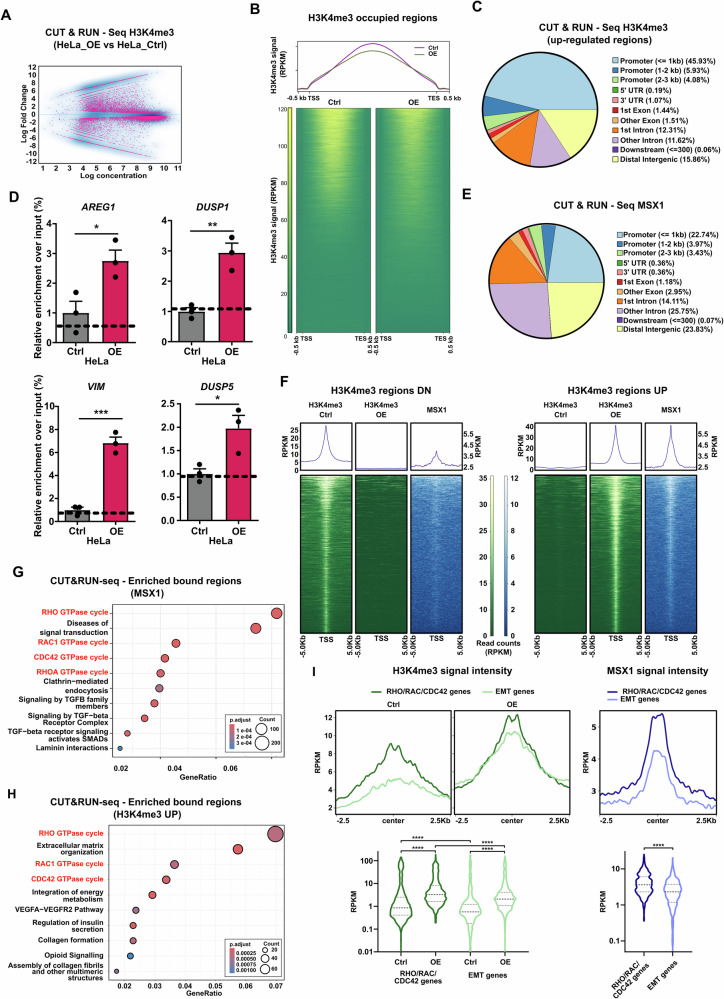
*MSX1* and H3K4me3 sequencing data reveals activation of RHO/RAC/CDC42 signaling. Genome-wide regulation of H3K4me3 regions upon MSX1 OE: results of a DiffBind analysis (**A**) and an aggregate plot overview (**B**). **C** ChIPseeker analysis showing the genomic distribution of up-regulated H3K4me3 regions in HeLa cells following MSX1 OE. **D** Validation of increased H3K4me3 occupancy at the promoter regions of EMT genes *AREG*, *DUSP1*, *VIM*, and *DUSP5* via ChIP-qPCR (*n* = *3* biological replicates). **E** ChIP-Seeker analysis depicting the genomic distribution of MSX1 binding sites (CUT&RUN data). **F** Aggregate plots showing MSX1 binding intensity at down-regulated (left panel) or up-regulated (right panel) H3K4me3 regions: Genomic regions that lose H3K4me3 occupancy upon MSX1 OE exhibit weak MSX1 binding, whereas regions gaining H3K4me3 show stronger MSX1 signal. **G** Pathway analysis reveals MSX1 binding sites are enriched for genes in the RHO GTPases RHO, RAC1 and CDC42 (RHO/RAC/CDC42) signaling pathways. **H** Pathway analysis identifies up-regulated H3K4me3 regions enriched for genes in the RHO/RAC/CDC42 signaling pathway. **I** Signal intensity analyses at genomic regions of EMT and RHO/RAC/CDC42 signature genes revealed a significant increase in H3K4me3 enrichment upon MSX1 OE (left panels). MSX1 binding was more pronounced in the regulatory regions of RHO/RAC/CDC42 signature genes compared to EMT genes (right panels). **A**, **B**, **C**, **H**, **I** H3K4me3 CUT&RUN data from *n* = 2 biological replicates. **E**–**G**, **I** MSX1 ChIP-seq data from *n* = 3 biological replicates. Statistical test: **D** Student’s *t* test; **I** Kruskal–Wallis test. *p < 0.05, **p ≤ 0.01, ***p ≤ 0.001, ****p-val ≤ 0.0001.

MSX1 is known as a transcription factor involved in various developmental processes by binding to specific DNA sequences in the promoter regions of target genes. MSX1 is particularly notable for its role in gene repression. It achieves this by recruiting co-repressors, such as members of the Groucho family, which interact with the MSX1 homeodomain [20]. Additionally, MSX1 can associate with the Polycomb Repressive Complex 2 (PRC2), which catalyzes the trimethylation of histone H3 at lysine 27 (H3K27me3), leading to chromatin compaction and gene silencing [21]. Although less well documented, MSX1 also has the capacity to activate gene expression in context-dependent manner [22]. To better understand the consequences of MSX1 binding on gene activation, we assessed changes of Histone 3 Lysine 4 trimethylation (H3K4me3), a histone mark enriched in active promoter regions, upon MSX1 OE by CUT&RUN (Fig. 3A). As expected from previous reports, most H3K4me3-occupied regions showed reduced occupancy (*n* = 3185 downregulated regions) upon MSX1 OE, consistent with the reported repressive activity of MSX1 (Fig. 3A, B). In contrast, a minority of gene regulatory regions showed increased H3K4me3 occupancy, especially at promoter regions (Fig. 3C). Notably, the upregulated genes associated with the EMT signature also exhibited increased H3K4me3 occupancy (Fig. 3D and Supplementary Fig. S2D). To better understand the consequences of MSX1 transcriptional activity in CC cells, we investigated its genome-wide binding activity using the CUT&RUN method. Subsequent analysis with ChIPseeker revealed that approximately half of the MSX1 binding sites localized to gene body regions, while ~25% of MSX1-bound sequences were found in distal intergenic regions, suggesting a potential role in enhancer activity. Additionally, ~25% of MSX1 binding sites were located at promoter or promoter-proximal regions of genes (Fig. 3E). To explore the functional implications of MSX1 binding at promoters, we analyzed MSX1 binding intensity at genomic regions that gained or lost H3K4me3 occupancy following MSX1 OE (Fig. 3F). Notably, regions that lost H3K4me3 occupancy upon MSX1 OE exhibited weak MSX1 binding, whereas regions with increased H3K4me3 occupancy showed strong MSX1 binding, suggesting a potential causal relationship between MSX1 binding and H3K4me3 dynamics (Fig. 3F). Building on the observed association between MSX1 binding and H3K4me3 modulation, we performed pathway enrichment analysis on MSX1-bound regions and genomic loci with increased H3K4me3 following MSX1-OE. Notably, both sets were significantly enriched for genes in RHO GTPase (RHO/RAC/CDC42) signaling pathway—key regulators of cell motility and invasion that cooperatively with EMT drive aggressive phenotypes in CC [23, 24] (Fig. 3G, H). Closer analysis of CUT&RUN data at regulatory regions of EMT and RHO/RAC/CDC42 genes revealed a significant increase in activating H3K4me3 marks in both gene signatures, along with strong MSX1 occupancy. Notably, MSX1 occupancy was higher at RHO/RAC/CDC42 genes than at EMT signature regions (Fig. 3I and Supplementary Fig. S2E, F). Collectively, these findings suggest that MSX1 may simultaneously activate EMT and RHO/RAC/CDC42 signaling in a cooperative manner, driving CC aggressiveness through coordinated regulation of cell motility and invasion.

### Active RHO/RAC/CDC42 signaling favors EMT in high *MSX1*-expressing cells

**Fig. 4:**
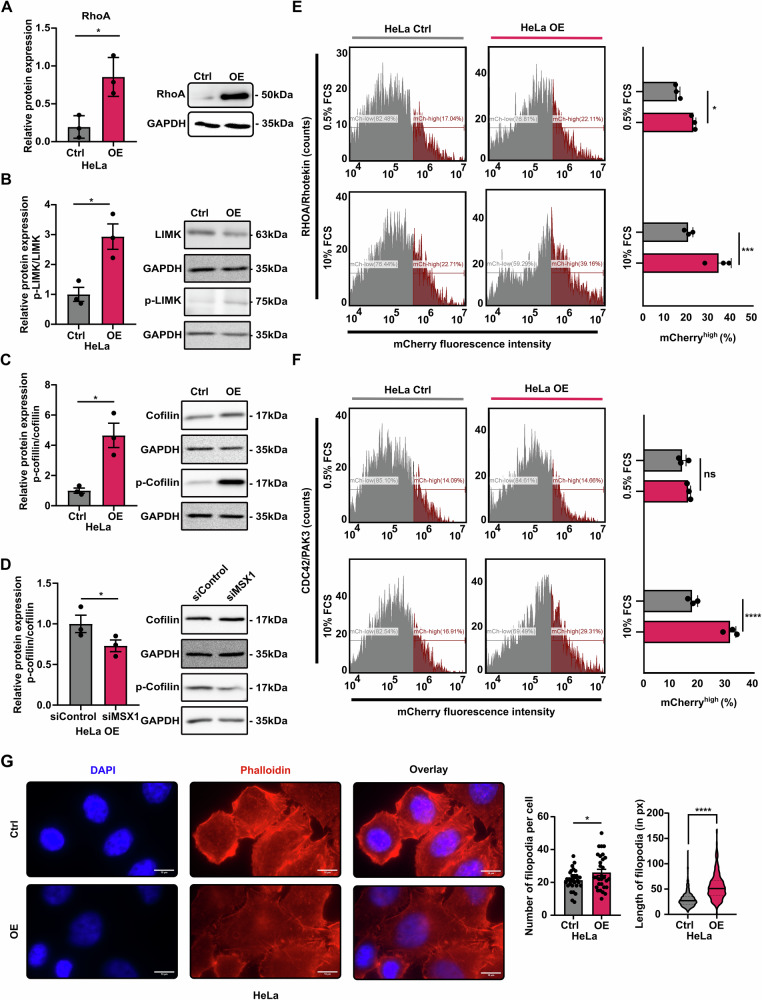
MSX1 increases RHO-GTPase activity. MSX1 OE in HeLa cells increases RHO GTPases downstream signaling: Western blot confirming elevated RhoA (**A**), p-LIMK (**B**) and p-Cofilin (**C**) levels. Densitometry quantifications are all normalized to respective loading control (GAPDH) levels. **D** MSX1 knockdown in OE cells reverts GTPase signaling, as assessed through Western blot for p-cofilin. FRET assay showing that MSX1 OE stimulates RHOA (**E**) and CDC42 (**F**) activation, as visualized by increased mCherry signal. Control (Ctrl) and OE cells were grown in 0.5% and 10% FCS to induce low or high RHO/RAC/CDC42 basal signaling activity, respectively. A quantification of the mCherry signal is shown in the respective right panel. **G** Phalloidin staining in HeLa control and MSX1 OE cells. (Scale bar: 10 µm). Statistical test: **A**–**D**, **G** (middle panel): Student’s *t* test; **E**, **F** one way ANOVA; **G** (right panel): Mann–Whitney. *p < 0.05, **p ≤ 0.01, ***p ≤ 0.001, ****p-val ≤ 0.0001. All experiments were performed in *n* = *3* biological replicates.

If RHO GTPase signaling acts cooperatively with EMT to promote aggressiveness, its inherent druggability may provide a viable strategy for disrupting this pro-invasive axis in CC. RHO-associated kinase 1 (ROCK1) and p21-activated kinase 3 (PAK3) activate LIM kinase (LIMK), which phosphorylates cofilin and inhibits its actin-depolymerizing function, promoting actin filament stabilization [25]. We first evaluated RHO/RAC/CDC42 pathway activity by monitoring phosphorylation of its key effectors. MSX1 OE resulted in upregulated RhoA and concomitant increases in p-LIMK and p-cofilin, supporting enhanced pathway signaling (Fig. 4A–C). In line with these findings, MSX1-KD reduced phosphorylation of cofilin (Fig. 4D). Direct assessment of RHO-GTPase activity by Western blot is not feasible, as these proteins are functionally regulated by GTP/GDP binding—states that cannot be distinguished by total protein detection, which is the primary readout of Western blotting. To assess potential activation of this signaling pathway by MSX1 OE, we employed a FRET-based approach described by Murakoshi and colleagues [26]. HeLa cells were transfected with either EGFP-tagged RHO and its effector mCherry-tagged RHOTEKIN, or EGFP-tagged CDC42 and its effector mCherry-tagged PAK3. To modulate RHO and CDC42 signaling activity, cells were cultured in 0.5% FCS (low basal activation) or 10% FCS (higher basal activation). As predicted by MSX1 binding activity, we observed increased FRET signals in MSX1-OE cells, indicating enhanced RHO-RHOTEKIN and CDC42-PAK3 interactions. Notably, activation of both RHO and CDC42 signaling branches was most pronounced under standard serum conditions (10% FCS) (Fig. 4E, F). In agreement with the observed transcriptional and signaling changes, MSX1-OE induced profound reorganization of the actin cytoskeleton in both HeLa and SiHa cells compared to controls. In HeLa cells, MSX1-OE led to a significant increase in both the number and length of filopodia (Fig. 4G). In contrast, SiHa cells exhibited a high baseline density of filopodia, and MSX1-OE further enhanced their length without increasing their number (Supplementary Fig. S2G). Together, these data demonstrate that MSX1 promotes a coordinated, RHO/RAC/CDC42- and EMT-driven program of cytoskeletal remodeling and cell motility, providing a mechanistic basis for targeting this axis in CC.

### Inhibition of RHO signaling attenuates MSX1-driven malignancy in cervical cancer cells

**Fig. 5:**
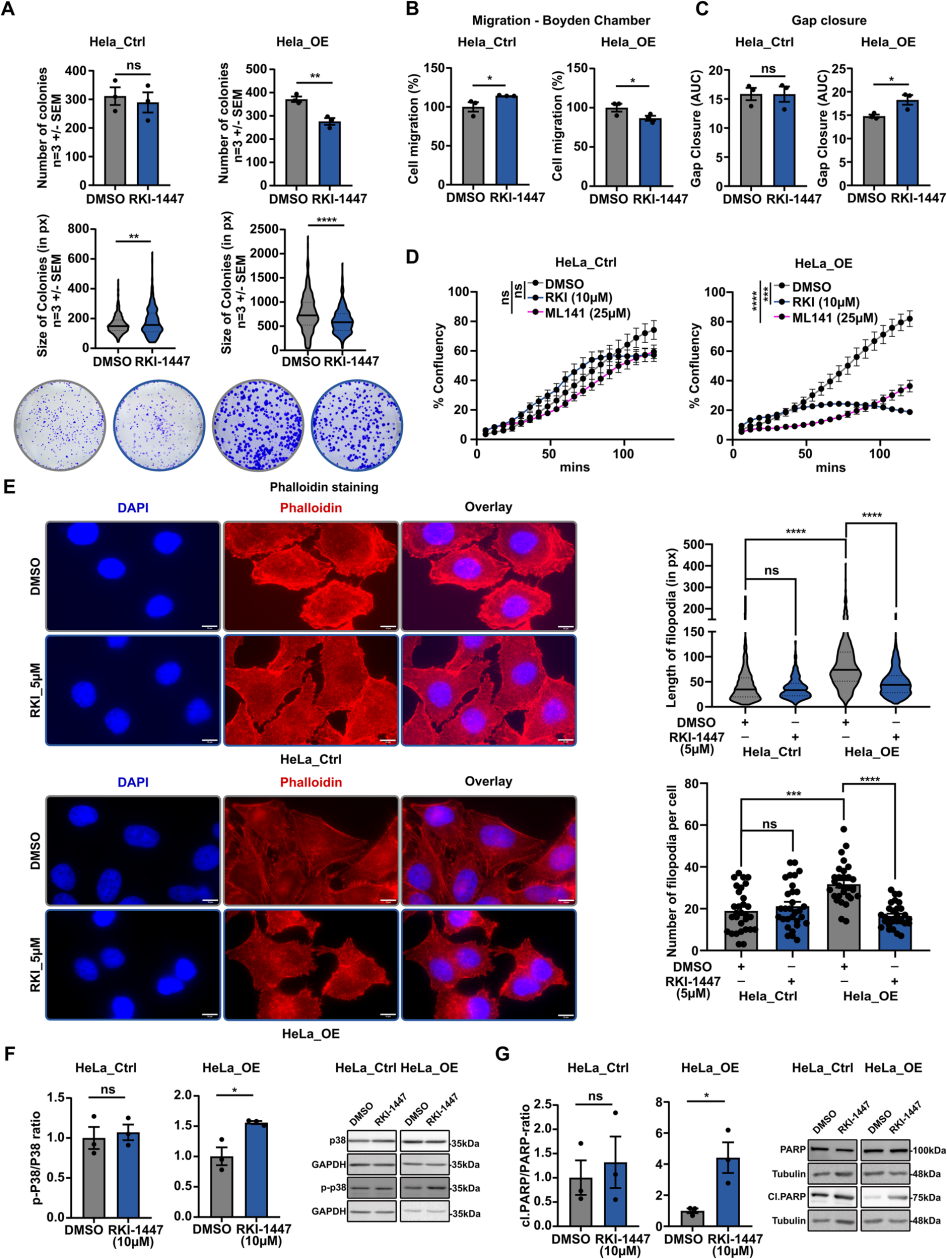
Interference with RHO signaling by RKI-1447 inhibitor reverts the MSX1-mediated aggressive phenotype in CC cells. **A** Colony formation assay in HeLa *MSX1*-overexpressing (OE) cells treated with RKI-1447 (ROCK1/2 inhibition, 1 µM). RKI-1447 treatment (10 µM) reverses the migratory phenotype of HeLa OE cells, as assessed by Boyden chamber (**B**) and gap closure (**C**) assays. **D** Growth kinetics of HeLa Ctrl (left panel) and OE (right panel) cells treated with either 10 µM RKI-1447 and 25 µM ML141. **E** Phalloidin staining showing reduction of filopodia structures in HeLa OE cells treated with RKI-1447 (5 µM). (Scale bar: 10 µm). Western blot analyses of stress kinase p38 and its phosphorylated form (p-p38) (**F**), and PARP and its cleaved fragment (**G**), in Ctrl and MSX1 OE cells treated with 10 μM RKI-1447. Densitometric quantification was performed and normalized to housekeeping proteins GAPDH (**F**) and tubulin (**G**). Statistical analysis: **A**–**C**, **F**, **G** Student’s *t* test; **D** AUC followed by Student’s *t* test; **E** Kruskal–Wallis test (Top panel) and One-way ANOVA (Bottom panel). *p-val < 0.05, **p ≤ 0.01, ***p-val ≤ 0.001, ****p-val ≤ 0.0001. All experiments were performed in *n* = *3* biological replicates.

To validate the therapeutic potential of targeting RHO GTPases signaling in MSX1-driven aggressiveness, we treated *MSX1*-overexpressing cells with the ROCK inhibitor RKI-1447 [27], which inhibits LIM-kinase phosphorylation. As expected, RKI-1447 reduced the clonogenic and migratory capacities of MSX1 OE cells, as assessed by Boyden chamber and wound healing assays, with minimal impact on control cells. (Fig. 5A–C and Supplementary Fig. S3A, B). Surprisingly, although MSX1 OE did not alter the growth kinetics of CC cells under standard conditions, inhibition of ROCK1 significantly impaired their proliferative capacity. These data support the notion that MSX1 OE specifically induces a novel dependency on RHO GTPase signaling. In addition, to validate this finding, we targeted the CDC42 branch of the pathway using the selective inhibitor ML141, which similarly reduced proliferation exclusively in *MSX1*-overexpressing cells, further supporting the emergence of a context-specific, MSX1-mediated vulnerability to RHO GTPase pathway inhibition (Fig. 5D). Additionally, RKI-1447 treatment effectively reversed the MSX1-driven increase in filopodia formation, with minimal impact on filopodia density in control cells, again indicating a selective dependence on RHO GTPase signaling in the context of MSX1 OE (Fig. 5E, Supplementary Fig. S3C). The pro-survival function of RHO GTPases—particularly through inhibition of apoptosis—has been well documented [28–30]. Given this, we hypothesized that MSX1-mediated upregulation of this pathway could serve as a compensatory mechanism to offset deficits in pro-survival signaling, in line with prior findings [15]. This dual role—supporting survival while facilitating EMT—suggests that the RHO/RAC/CDC42 network may serve as a molecular bridge between stress resistance and phenotypic plasticity. This hypothesis was supported by the observation that RKI-1447 treatment specifically induced activation of the stress kinase p38, as evidenced by increased phosphorylation (p-p38), in *MSX1*-overexpressing cells (Fig. 5F). Concomitantly, apoptosis marker cleaved PARP was upregulated exclusively in an MSX1-dependent manner, with no detectable increase observed in control cells (Fig. 5G). Notably, basal levels of cleaved PARP were lower in *MSX1*-expressing cells compared to controls, suggesting a potential MSX1-dependent suppression of apoptosis. Collectively, these findings identify MSX1-driven dependency on RHO GTPase signaling as a critical regulator of both aggressive behavior and survival, underscoring its potential as a therapeutic target in context-specific cancers.

### FOS links RHO signaling to MSX1-driven tumor cell aggressiveness

To uncover molecular mediators linking RHO/RAC/CDC42 activation to MSX1-driven phenotypes, we aimed to identify critical downstream effectors involved in transcriptional reprogramming. Among potential downstream mediators, FOS stood out as a transcription factor associated with RHO/RAC/CDC42 activity. It has been linked to anti-apoptotic functions [31] and cooperates with EMT-inducing pathways [32]. FOS, a component of the AP-1 complex, plays a critical role in cell proliferation, differentiation, and survival, and exhibits context-dependent tumorigenic or anti-tumorigenic activity in various cancers [33].

**Fig. 6:**
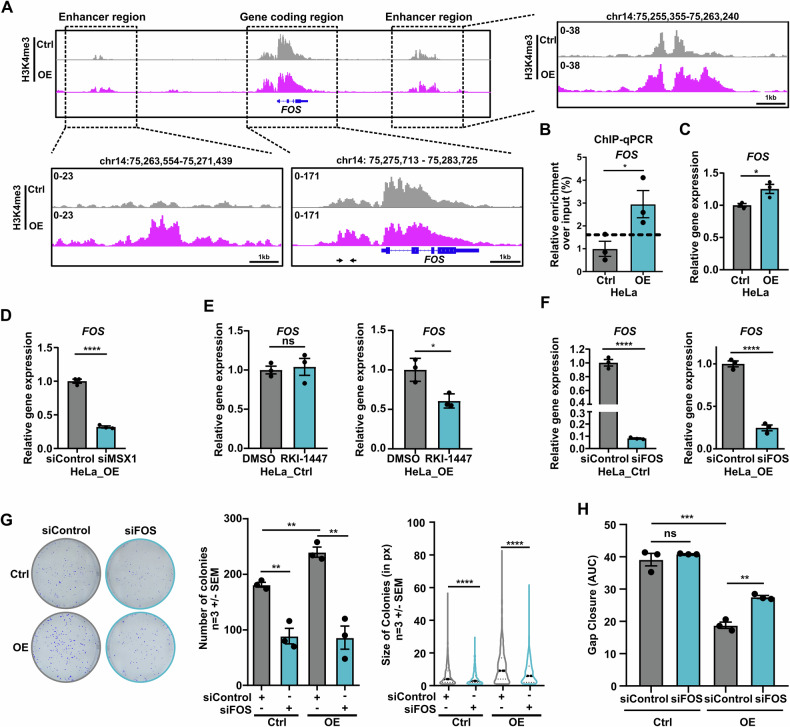
MSX1-mediated aggressive phenotype require RHO signaling induction of FOS. **A** IGV profile showing increased H3K4me3 occupancy at the promoter and enhancer regions of the *FOS* gene in HeLa MSX1 OE cells. **B** Validation of elevated H3K4me3 enrichment at the *FOS* promoter via ChIP-qPCR. The localization of qPCR primers is indicated by black arrows in the lower middle panel of (**A**). **C** RT-qPCR confirming increased *FOS* expression in HeLa OE cells. **D** MSX1 knockdown reversed the elevated *FOS* expression levels in HeLa *MSX1*-overexpressing cells, as determined by RT-qPCR. **E** Treatment of HeLa OE cells with 10 µM RKI-1447 reduces *FOS* expression levels, as assessed by RT-qPCR. **F** Validation of FOS knockdown (KD) via RT-qPCR in HeLa control and OE cells. **G** siRNA-mediated FOS KD in HeLa ctrl and OE cells reduces colony formation capacity, with a more pronounced effect in MSX1-positive cells. **H** FOS knockdown impairs motility of HeLa OE cells but not control cells, as assessed in a gap closure assay (AUC). Statistical analysis: **B**–**H** Student’s *t* test; **G** (right panel): Mann–Whitney test. **H** AUC followed by Student’s *t* test. *p < 0.05, **p ≤ 0.01, ***p ≤ 0.001, ****p ≤ 0.0001. **B**–**H** experiments were performed in *n* = *3* biological replicates.

Strikingly, ChIP-Seq analysis for H3K4me3 revealed enriched peaks at regulatory regions of the *FOS* gene in MSX1-OE cells (Fig. 6A), which were further validated by ChIP-qPCR (Fig. 6B). Consistently, *FOS* expression was elevated in MSX1 OE cells, as demonstrated by mRNA-Seq and qPCR (Fig. 6C). In contrast, the siRNA- mediated MSX1 knockdown (KD) reduced the *FOS* levels in *MSX1*-overexpressing cells (Fig. 6D). To assess the role of RHO/RAC/CDC42 signaling in *FOS* upregulation, we performed qPCR on control and *MSX1*-overexpressing HeLa cells treated with RKI-1447. Indeed, treatment reduced *FOS* levels (Fig. 6E). To determine whether increased *FOS* expression contributes to MSX1-mediated tumor cell aggressiveness, we performed siRNA-mediated FOS KD in control and OE cells (Fig. 6F). FOS depletion reduced clonogenicity, with a pronounced effect in OE cells (Fig. 6G). Furthermore, FOS KD did not affect the migratory potential of control cells but significantly impaired motility in HeLa OE cells (Fig. 6H, Supplementary Fig. S3D). Overall, our data demonstrate that RHO signaling inhibition reverses MSX1-dependent aggressiveness, particularly in clonogenic and migratory properties.

## Discussion

CC, despite being highly preventable and treatable when detected early, remains a leading cause of cancer-related mortality among women in many parts of the world [2]. This underscores the need for deeper research into its molecular mechanisms to develop effective therapeutic strategies. Recent studies have highlighted the role of transcription factor MSX1 in various gynecological cancers, but its role in CC has been less explored. With the present study, we sought to close this gap focusing on its potential implications for targeted therapies. To our surprise, and in contrast to its well-documented tumor-suppressive role in certain cancers—including breast cancer, endometrial cancer, CC, ovarian carcinoma, and esophageal carcinoma [14, 15, 18, 34–36],—our study identifies MSX1 as a potent tumor-promoting factor in CC. This functional duality underscores the context-dependent nature of MSX1, which can act as either an oncogene or a tumor suppressor depending on the cellular and molecular environment. Yue et al. reported frequent DNA methylation at the *MSX1* promoter in CC, proposing that epigenetic silencing of MSX1 contributes to tumor cell fitness by releasing a brake on proliferation and survival. In contrast, our data reveal a strikingly opposite role in CC: MSX1 is aberrantly expressed and drives tumor aggressiveness by coordinately inducing EMT and activating the RHO/RAC/CDC42 signaling axis. This oncogenic function is further supported by evidence from colorectal cancer, melanoma, and bone cancer, where *MSX1* overexpression correlates with cancer aggressiveness [37–39]. Together, these findings highlight the dual nature of MSX1 and emphasize that its role in tumorigenesis is highly tissue-specific, with implications for both diagnosis and targeted therapy. Our present investigations improved our understanding of *MSX1* expression in normal and pre-cancerous lesions, allowing us to differentiate between glandular and squamous epithelial tissue of the cervix. We demonstrated that glandular epithelial cells of the endocervix are strongly MSX1 positive while squamous epithelial cells of the ectocervix are MSX1 negative. Squamous CC originating from the MSX1 negative ectocervix tissues is the most frequent CC. Therefore, it is reasonable to posit that previous reports on *MSX1* loss during cervical tumorigenesis might reflect a comparison of these lesions with normal tissue biopsies harboring both squamous and glandular cell types. Indeed, the present study revealed that MSX1 is reactivated in pre-cancerous squamous cervical lesions, and correlates with poorer survival outcomes and increased severity of cervical intraepithelial neoplasia (CIN). Further, in vitro experiments demonstrated that MSX1 OE in various CC cell lines (HeLa and SiHa) enhances their aggressiveness (e.g., clonogenicity, migration). These data partially contradicting the observations made by Yue et al. [15], might be the result of different strategies to overexpress *MSX1*. Indeed, increasing evidences argue for potential tumor supportive functions of MSX1 in colorectal cancer and melanoma cells [37, 38, 40]. Together with our observation in pre-cancerous patient material, our findings suggest that MSX1 plays a critical role in promoting tumor aggressiveness in CC.

Mechanistically, we provide evidence that MSX1 induces EMT, a process associated with tumor aggressiveness, metastasis, and therapy resistance [41]. EMT is characterized by the loss of epithelial markers and the acquisition of mesenchymal features, enabling cancer cells to migrate and invade surrounding tissues. MSX1 OE was found to upregulate EMT-associated genes such as *AREG*, *DUSP1*, *DUSP5*, *ACTA2*, and *VIM*. These genes are involved in cell migration, survival, and cytoskeletal reorganization, which are hallmarks of EMT. Our data support the observations by Heppt and colleagues in cells, where MSX1 OE induced EMT phenotypes and invasion in melanoma cells. Similarly to our results, MSX1 modulation did not influenced melanoma cell proliferation [37]. We leveraged whole transcriptome and genome-wide binding studies to better understand the transcriptomic activity of MSX1 potentially leading to EMT induction in CC. Concurrent with EMT induction, we found that MSX1 OE also stimulated the RHO/RAC/CDC42 signaling cascade. RHO GTPases regulate cytoskeletal dynamics, cell migration, and proliferation [42]. MSX1 OE led to increased activity of RHOA and CDC42, resulting in the reorganization of stress fibers and induction of filopodia, which are essential for cell migration. The activation of RHO signaling also upregulates downstream targets such as FOS, a transcription factor involved in cell proliferation and survival [33]. These findings highlight the molecular mechanisms by which MSX1 promotes EMT and tumor aggressiveness in CC. The identification of MSX1 as a tumor-promoting factor in CC opens new avenues for targeted therapies. Our study suggests that inhibiting RHO signaling could be an effective strategy to revert the aggressive phenotype induced by MSX1. ROCK inhibitors, which target the RHO signaling pathway, were shown to reduce clonogenicity, migration, and cytoskeletal changes in *MSX1*-expressing CC cells. These findings are consistent with previous reports demonstrating the efficacy of ROCK inhibitors in other cancers, such as breast and ovarian cancer [43, 44].

In conclusion, the present study significantly advances our understanding of MSX1’s role in CC, identifying it as a tumor-promoting factor that drives EMT and activates RHO/RAC/CDC42 signaling. These pathways act cooperatively to enhance tumor aggressiveness by coordinately regulating cell motility, invasion, and stress adaptation. The findings have important implications for the development of targeted therapies, particularly those targeting RHO signaling or MSX1 itself. However, further research is needed to explore the clinical potential of these therapeutic strategies and to investigate the role of MSX1 in other cancer types. The study underscores the importance of continued research into the molecular mechanisms of CC to improve treatment outcomes for patients.

## Methods

### Cell culture, transfections and functional assays

HeLa (ATCC ® CCL-2 ^TM^) and SiHa (ATCC ® HTB-35 ^TM^) cells were cultivated in Minimum Essential Medium (MEM) (Biowest) supplemented with 10% fetal bovine serum (Biowest), 1x penicillin/streptomycin (Gibco) at 37 °C with 5% CO2. The Sleeping Beauty Vector system was used to stably integrate the *MSX1* gene (HeLa, SiHa) (VectorBuilder GmbH) or *MSX1* tagged with *FKBP12* (HeLa) (VectorBuilder GmbH) into the genome of the cells by the usage of a transposase (Supplementary Table S1). The plasmid transfections were performed using Turbofect (Thermo Fisher Scientific) in OptiMEM GlutaMAX (Gibco) according to the manufacturer’s protocol. FACS sorting (BD FACSAria ™ II Cell Sorter; BD Life Sciences) was performed using GFP as a selection marker for MSX1-positive cells after 3 days. The positive cells were grown as single-cell clones or in a pool and used in three biologicals for subsequent experiments. siRNA transfections were performed using DharmaFECT1 (Dharmacon) in OptiMEM GlutaMAX (Gibco) according to the manufacturer’s protocol. A list of siRNAs used in this study is provided in Supplementary Table S2. Proliferation kinetics was recorded using a Celigo® S imaging cytometer (Nexcelom Bioscience LLC). For endpoint analysis of proliferation kinetics and colonies for clonogenic assays, cells were fixed with methanol (J.T.Baker) for 20 min, stained with 0.25% crystal violet in 20% methanol (J.T.Baker) for 20 min, washed, air dried and scanned with an Epson Perfection V850 PRO. Details of used functional assay protocols are provided in the supplementary data. The results from the analysis of functional assays were plotted using GraphPad Prism v8.0.1. All the experiments were performed with mycoplasma free cells.

### Gap closure assay

Approximately 70,000–100,000 cells were seeded into two-well inserts with a 500 µm gap (Ibidi), which were placed in a 24-well plate. Once the cells had adhered overnight, the wells within the inserts were washed with PBS, and the inserts were removed, leaving a gap in the cell monolayer. The 24-well plate was then washed with PBS and replenished with complete medium with or without RKI treatment. The gaps were scanned under a microscope every 12 h until the gaps in the control condition had closed. The area of the gaps was analyzed using ImageJ and normalized to the initial area at day 0. The results of the analysis were plotted using GraphPad Prism v8.0.1.

### Boyden chamber assay

Approximately 80,000 cells in 250 µL of low-serum (0.5% FBS) medium were seeded into the upper chamber of pre-equilibrated semipermeable membrane inserts (8 µm pores, Sarstedt). One hour post-seeding, the medium in the lower chamber was replaced with complete medium (10% FBS). For RKI treatment, cells were seeded into the upper chamber of pre-equilibrated semipermeable membrane inserts in complete medium (10% FCS) and allowed to adhere overnight. Next day, medium was replaced with 250 µl of low-serum (0.5% FBS) in upper chamber and 750 µl of complete medium (10% FBS) in lower chamber, both supplemented with RKI. After incubation for 24–48 h at 37 °C, the inserts were washed with PBS and fixed in 4% paraformaldehyde (PFA, 500 µL) for 10 min, followed by another PBS wash. Cells on the upper layer of the insert were carefully removed using a cotton swab. The inserts were then stained with Crystal Violet for 10 min (500 µL in the lower chamber), and scans of the plates were analyzed using ImageJ. The results of the analysis were plotted using GraphPad Prism v8.0.1.

### Invasion assay

250 cells in 20 μl were seeded in hanging drops onto the lid of a 24-well plate. The plate itself was filled with PBS to prevent the drops from drying. After 7 days spheres were seeded in 30 μl of collagen-matrigel (7:3 ratio) matrix in a 24-well plate. Organoid medium (DMEM/F12, 1% P/S, 1X ITS, 2.5 nM FGF2 and 1X B27) was added after the matrix polymerized (~1 h after seeding at 37 °C). Images were taken every day with a motorized inverted microscope (Olympus IX83).

### Fluorescence resistance energy transfer (FRET) assay

For measurement of RHO or CDC42 activity, HeLa cells were co-transfected with two plasmids namely: either EGFP-tagged RHO and Rhotekin tagged with an mCherry, or EGFP-tagged CDC42 and PAK3 tagged with an mCherry, respectively (Plasmids are listed in Supplementary Table S1) [26]. The plasmid transfections were performed using TurboFect (Thermo Fisher Scientific) in OptiMEM GlutaMAX (Gibco) according to the manufacturer’s protocol. Single-plasmid-transfected cells (1× EGFP plasmid, 1× mCherry plasmid) and non-transfected cells were used as control. After 3 days, transfected cells were trypsinized and resuspended in 1 ml of PBS supplemented with 1% FCS. All the samples were measured with the FACS CytoFlex S system (Beckman Coulter). First, transfection efficiency was checked, measuring GFP emission with the FITC filter (525 nm), whereas mCherry emission was measured with the PI filter (610 nm). For FRET analyses, samples were only excited with the blue laser (488 nm) and FRET signal was measured at 610 nm. The living cell population was determined using forward and side scatter (FSC and SSC).

### RNA isolation and real-time quantitative PCR (RT-qPCR)

RNA isolation, cDNA synthesis, and RT-qPCR were performed as previously described [45]. The primer sequences used in this study are provided in Supplementary Table S3. Detailed protocols of RNA extraction and cDNA synthesis are provided in the Supplementary Data. Results were graphed using GraphPad Prism v8.0.1.

### Protein analysis

Protein extraction and quantification were performed according to the standard protocols of the lab [45]. Samples were subsequently analyzed by Western Blot. Detailed descriptions are provided in the supplements. Full and uncropped Western blots are available in the supplemental data.

### mRNA library preparation and data analysis

mRNA-seq libraries were prepared following a previously established protocol [46] 48 h post-transfection using the TruSeq® RNA Library Prep Kit v2 (Illumina) as per the manufacturer’s guidelines. Samples were sequenced (single-end 50 bp) on a HiSeq4000 (Illumina) at the NGS Integrative Genomics Core Unit (NIG) of the University Medical Center Göttingen (UMG). Sequencing (single-end, 50 bp reads) was conducted on a HiSeq4000 (Illumina) at the NGS Integrative Genomics Core Unit (NIG) of the University Medical Center Göttingen (UMG). Data processing and analysis were performed using the Galaxy platform hosted by the Gesellschaft für Wissenschaftliche Datenverarbeitung GmbH Göttingen (GWDG). Raw reads were trimmed to remove the first 11 nucleotides (FASTQ Trimmer, v1.1.1) and aligned to the hg38 reference genome via HISAT2 (v2.1.0+galaxy5) [47]. Gene-level read counts were quantified using featureCounts (v1.6.3+galaxy2) [48], and differential gene expression analysis was conducted with DESeq2 (v2.11.40.6+galaxy2) [49]. Only genes exhibiting a basemean ≥10 considered expressed. Pathway enrichment analysis was performed using Gene Set Enrichment Analysis (GSEA, v4.1.0; https://www.gsea-msigdb.org/gsea/msigdb).

### CUT&RUN library preparation and data analysis

Genome wide distribution of H3K4me3 was assessed by CUT&RUN technology using the ChIC/CUT&RUN Assay Kit from Active Motif Kit (#53180, Active Motif) following the manufacturer’s instruction. The antibodies used for in this study are listed in Supplementary Table S4. Next-generation sequencing library was prepared using the KAPA Hyper Prep Kit (KR0961–v6.17) according to manufacturer’s instructions and samples were sequenced (paired-end 100 bp) on a DNBSEQ-G400 (MGI) at the BGI sequencing facility (BGI Poland). Processing of sequencing data was performed in the Galaxy environment (galaxy.gwdg.de). Briefly, reads were mapped to the hg38 reference genome assembly using Bowtie2 (version 2.3.4.2). PCR duplicates were removed using the RmDup tool (version 2.0.1). The bamCoverage tool (version 3.2.0.0.0) was utilized to generate normalized coverage files using reads per kilobase per million (RPKM) as a normalizing method. Peak calling was performed using the MACS2 callpeak (version 2.1.1.20160309.0), and computeMatrix and plotHeatmap (version 2.5.1.1.0) to generate aggregate plots and heatmaps, respectively. Occupancy profiles were visualized using the Integrative Genomics Viewer (IGV 2.14.0). Peak annotation and gene signature enrichment were performed using ChIPseeker (v1.30.0) [50] in the R environment (version 4.4.1) with RStudio (2024.04.2).

### Immunofluorescence staining

100,000 cells were seeded on coverslips in 6-well plate, washed with PBS, fixed in 4% PFA for 15 min, then permeabilized in 1% Triton in PBS for 5 min, and finally blocked in 0.5% BSA in PBS for 60 min. The coverslips were incubated overnight with the primary antibodies (see Supplementary Table S4) diluted in 0.5% BSA in PBS (1:100) in a humid chamber at 4 °C. The coverslips were then washed with PBS-T (PBS + 0.1% Tween20) and incubated with secondary antibodies (1:400, Supplementary Table S5) and DAPI (1:1000) diluted in 0.5% BSA in PBS for 60 min at room temperature in the dark humid chamber. In the case of Phalloidin staining, Phalloidin (1:1000), and DAPI (1:1000) were diluted in 0.5% BSA in PBS and incubated for 30 min at room temperature. Mowiol-488 was used to mount the coverslips.

### Immunohistochemistry staining

Paraffin-embedded tissue sections were deparaffinized through a descending ethanol series: 20 min in xylene, followed by 5 min each in 100%, 90%, and 70% ethanol. Antigen retrieval was performed by heating the slides in 1 mM citrate buffer (pH 6.0) for 10 min at ~300 W using a microwave oven. Endogenous peroxidase activity was quenched by incubating the sections with 3% hydrogen peroxide in PBS for 45 min. Non-specific binding was blocked by incubating the slides with 5% bovine serum albumin (BSA) in PBS containing 1:1000 donkey serum for 1 h at room temperature. Sections were then incubated overnight at 4 °C with a primary antibody against MSX1 (1:100 dilution in PBS). After washing with PBS, the slides were incubated with a secondary antibody (anti-donkey rabbit IgG, 1:1000) for 90 min at room temperature. For signal amplification, extravidin-peroxidase (1:1000 in PBS) was applied and incubated for 120 min. Staining was developed using 3,3’-diaminobenzidine (DAB) under microscopic supervision for 10 min. Nuclear counterstaining was performed with hematoxylin, followed by thorough rinsing with running tap water. Following staining, sections were dehydrated through ascending ethanol concentrations (5 min each in 70%, 90%, and 100% ethanol), cleared in xylene (20 min), and mounted with aqueous mounting medium. Coverslips were applied, and slides were left to dry overnight before imaging.

## Supplementary information

SUPPLEMENTAL MATERIAL

Original Data

## Data Availability

NGS data have been deposited at ArrayExpress (https://www.ebi.ac.uk/arrayexpress/) under the accession numbers E-MTAB-15395 (mRNA-seq) and E-MTAB-15389 (CUT&RUN-seq).
