# Supplementary material for: Identification of tumor-promoting functions of the Homeobox family transcription factor MSX1 in cervical cancer
Source: Cell Death Discov. 2026 Jun 5;12:270. doi: 10.1038/s41420-026-03191-y (PMC13241535; doi:10.1038/s41420-026-03191-y)
Supplement: Supplementary file 1 — SUPPLEMENTAL MATERIAL [file 41420_2026_3191_MOESM1_ESM.docx]

**Supplementary material**

**Identification of tumor-promoting functions of the Homeobox family transcription factor MSX1 in cervical cancer.**

**Paulina Brücker^1§^, Svenja Horn^1§^, Shaishavi Jansari^1§^, Marianna Kombou^1^, Simon Eppich^2^, Udo Jeschke^2,3^, Samy Hakroush^4^, Stefan Küffer^4^, Sonja Fritzsche^1^, Florian Wegwitz^1#^, Julia Gallwas^1#^**

*^1^Department of Gynecology and Obstetrics, University Medical Center Göttingen, Göttingen, Germany.*

*^2^Department of Obstetrics and Gynecology, University Hospital, Ludwig Maximilians University, Munich, Germany.*

*^3^Gynecology, Faculty of Medicine, University of Augsburg, Augsburg, Germany.*

*^4^Institute of Pathology, University Medical Center Göttingen, Göttingen, Germany.*

§ and #: these authors contributed equally

# Address correspondence to [fwegwit@gwdg.de](mailto:fwegwit@gwdg.de) (F.W.)

**Supplementary Methods**

***Functional assays***

All experiments were performed in biological triplicates. The results were plotted with GraphPad Prism v8.0.1.

*Proliferation kinetics*: For MSX1 overexpression (MSX1-OE) experiments, 12,000 cells were seeded per well in a 24-well plate. Cell confluency was monitored every 48 h using a Celigo® S imaging cytometer (Revvity, Inc.) until control cells reached 100% confluence. For proliferation assays under pharmacological inhibition, HeLa C (control) and HeLa M (MSX1-overexpressing) cells were seeded at 2,500 cells per well in 96-well plates. Proliferation was tracked continuously using the CellCyte 3™ automated cell imaging system (Echo), with images acquired every 6 h to generate real-time proliferation curves.

*Clonogenic assay*: 500 and 1.000 cells were seeded in a 6-well plate. 7-10 days after seeding, colonies were washed with PBS, fixed with methanol for 20 min, stained with 0.25 % crystal violet in 20% methanol for 20 min and washed with tap water. Finally, stained colonies were scanned using an EPSON perfection V850 PRO scanner. The number and size of colonies were analyzed using ImageJ.

***RNA isolation and real-time quantitative PCR (RT-qPCR)***

For stable MSX1 overexpression, 100,000–200,000 cells were seeded per well in a 6-well plate and harvested for RNA isolation 48 hours post-seeding. For knockdown experiments, 200,000–500,000 cells were reverse transfected with the respective siRNAs in 6-well plates, and RNA was extracted 72 hours post-transfection. For inhibitor treatments, 200,000 cells were seeded per well. Inhibitor treatment was initiated 24 hours after seeding, and cells were harvested for RNA extraction 48 hours after treatment. For RNA extraction, cells were washed with PBS and lysed in 500 µl EXTRAzol (EM30-100, BLIRT). Lysates were then collected and RNA was extracted, as previously described (Prokakis et al., 2021). Reverse transcription of 1 µg RNA was performed using M-MuLV reverse transcriptase (GeneON) with random primers according to the manufacturer’s instructions. The expression of specific genes was finally estimated by quantitative real-time PCR using a CFX Connect™ Real-Time System (Bio-Rad). Gene expression levels were normalized relative to the *RPLP0* housekeeping gene (all RT-qPCR experiments). RT-PCR program: 1x 2 min/95 ^o^C, [10 sec-95 ^o^C followed by 1x 30 sec/60°C] for a total or 40 cycles. Primers (listed in Table S3) were designed using the online tool https://www.ncbi.nlm.nih.gov/tools/primer-blast/ and were ordered from Sigma-Aldrich (Germany). The results were finally plotted with GraphPad Prism v8.0.1.

**Protein isolation and western blot analyses**

For stable MSX1 overexpression (MSX1-OE) experiments, 200,000 cells were seeded per well in 6-wells and harvested for protein extraction 48 h post-seeding. For knockdown experiments, 200,000 cells were reverse transfected with siRNAs and harvested 72 h post-transfection. For inhibitor treatments, 80,000 cells were seeded, and treatment was initiated 24 h later, with protein extraction performed 72 h after the start of treatment. Cells were washed once with PBS, and 300 μl of RIPA buffer (10 mM Tris-Cl pH 8, 1 mM EDTA, 1% v/v Triton X-100, 0.1% sodium deoxycholate, 0.1% SDS, 140 mM NaCl, supplemented with protease and phosphatase inhibitors (1 μM activated orthovanadate, 10 mM β-glycerophosphate disodium salt hydrate, 10 mM Pefablock, 10 mM N-Ethylmaleimide, 1 mM Aprotinin/Leupeptinin, 1mM NaF, 1 μM iodoacetic acid) was added to each well (6 well plate). After 10 min incubation on ice, cells were scraped and lysates were sonicated for ten cycles (each 30s ON/ 30s OFF) using a Bioruptor (Diagenode). Protein concentration of the cell lysates was assessed with the BCA method (Pierce). 6x Laemmli buffer (375 mM Tris/HCl, 10% SDS, 30% glycerol, 0.02% bromophenol blue, 9.3% DTT) was added to each lysate and cooked at 95°C for 5 min before protein separation with a 10 to 12% polyacrylamide gel. Proteins were transferred to nitrocellulose or PVDF membrane (0.45 µm pore, Immobilon, Millipore), blocked with 5% skimmed milk in TBS-T for 1h and incubated with primary antibody overnight at 4°C. The day after, the membrane was washed with TBS-T, and incubated for 1h with a secondary antibody at room temperature. After a final wash step, protein detection was achieved with WESTAR ECL Substrates (Cyanagen) in an Intas Chemostar Imager (Intas Science Imaging). Primary and secondary antibodies used in this study are listed in Table S4 and S5 respectively.

**Supplementary Tables**

**Table S1: Plasmids used for Transfection in this study.**

| **Plasmid Name** | **Plasmid Number** | **Company** |
| --- | --- | --- |
| SB-MSX1 | VB201119-1011fge | VectorBuilder GmBH, Neu-Isenburg, Germany |
| SB-Transposase | VB180507-1151nqp | VectorBuilder GmBH, Neu-Isenburg, Germany |
| mEGFP-RHOA-C1 | #29674 | Addgene Europe, Teddington, UK |
| mCherry-RHOTEKIN(8-89)-mCherry-C1 | #29675 | Addgene Europe, Teddington, UK |
| mEGFP-Cdc42-C1 | #29673 | Addgene Europe, Teddington, UK |
| mCherry-Pak3(60-113)/S74A/F84A-mCherry-C1 | #29676 | Addgene Europe, Teddington, UK |

**Table S2: List of siRNA’s used in this study.**

| **Gene** | **siRNA** | **Cat. Number** |
| --- | --- | --- |
| Non-Τargeting #5 | UGGUUUACAUGUCGACUAA | D-001210-05-50 Dharmacon |
| c-FOS | #1 GGGAUAGCCUCUCUUACUA | SO-2001-03 Dharmacon |
|  | #2 GAACAGUUAUCUCCAGAAG | SO-2001-03 Dharmacon |
|  | #3 GGAGACAGACCAACUAGAA | SO-2001-03 Dharmacon |
|  | #4 AGACCGAGCCCUUUGAUGA | SO-2001-03 Dharmacon |
| MSX1 | #1 AAACACAAGACGAACCGUA | MQ-011733-01-0005 Dharmacon |
|  | #2 AGAAGAUGCGCUCGUCAAA | MQ-011733-01-0005 Dharmacon |
|  | #3 CAAGGCAAAGAGACUACAA | MQ-011733-01-0005 Dharmacon |
|  | #4 GUGUCAAAGUGGAGGACUC | MQ-011733-01-0005 Dharmacon |

**Table S3: RT-qPCR and CHIP-qPCR primers used in this study.**

| **Gene name** |  | **Sequence** | **Species** | **Reference** |
| --- | --- | --- | --- | --- |
| ***AREG*** | **Forward** | CGGGAGCCAACTATGACTAC | Human | This study |
|  | **Reverse** | TCACTTTCCGTCTTGTTTTGG |  |  |
| ***DUSP1*** | **Forward** | CCAACCATTTTGAGGGTCAC | Human | This study |
|  | **Reverse** | GACAAACACCCTTCCTCCAG |  |  |
| ***DUSP5*** | **Forward** | CAGGCCAGCTTATGACCAGG | Human | This study |
|  | **Reverse** | TCGCACTTGGATGCATGGTA |  |  |
| ***FOS*** | **Forward** | CCGGGGATAGCCTCTCTTAC | Human | This study |
|  | **Reverse** | GTGGGAATGAAGTTGGCACT |  |  |
| **MSX1** | **Forward** | AGAAGATGCGCTCGTCAAAG | Human | This study |
|  | **Reverse** | GGCTTACGGTTCGTCTTGTG |  |  |
| ***RPLP0*** | **Forward** | CCAGTGTACAGCAGCCTGAA | Human | This study |
|  | **Reverse** | ACTTGGGCCGGTATTTCTTC |  |  |
| ***Vimentin*** | **Forward** | TACAGGAAGCTGCTGGAAGG | Human | This study |
|  | **Reverse** | ACCAGAGGGAGTGAATCCAG |  |  |
| ***ACTA2*** | **Forward** | TTACGAGTTGCCTGATGGGC | Human | This study |
|  | **Reverse** | TCTCCTTCTGCATTCGGTCG |  |  |
| **AREG_qChIP** | **Forward** | AATGTTGATGAGGCCCCTGG | Human | This study |
|  | **Reverse** | CCCTTCCCACCCATTTCTGT |  |  |
| **DUSP1_qChIP** | **Forward** | ACAGGGGCAAAGTCAAGGAC | Human | This study |
|  | **Reverse** | TCGCCTTATCCATGAGCACC |  |  |
| **DUSP5-qChIP** | **Forward** | ACAGGGGCAAAGTCAAGGAC | Human | This study |
|  | **Reverse** | CCGAGTTGAGGTTGACGTTG |  |  |
| **FOS-qChIP** | **Forward** | CCCAGCAGTCGAGGTATTCC | Human | This study |
|  | **Reverse** | TCAGTTCGGGATGACAAGGC |  |  |
| **Vimentin_qChIP** | **Forward** | CTCCTCTGTCCCCACACATT | Human | This study |
|  | **Reverse** | CTTCCCCTCCTTCCTTCTCC |  |  |

**Table S4: Primary Antibodies**

| **Antibody** | **WB (dilutions)** | **IF**  **(dilutions)** | **ChIP/CUT & RUN** | **cat. number, company** |
| --- | --- | --- | --- | --- |
| Anti-Actin RHOdamin hFAB | (1:5000) |  |  | #12004164,BioRad |
| MSX1 | (1:1000) | (1:100) | 1 µg | #5378S,Cell Signaling |
| Vimentin | (1:1000) | (1:100) |  | #Ab92547,Abcam |
| GAPDH | (1:1000) |  |  | #5174S,Cell Signaling |
| RHO-A | (1:1000) |  |  | Cat.# ARH03 Cytoskeleton |
| P-Cofilin | (1:1000) |  |  | #77G2, Cell Signaling |
| H3K4 |  |  | 1 µg | #91263, ActiveMotif |
| IgG |  |  | 1 µg | #2729S,Cell Signaling |
| Cofilin1 | (1:1000) |  |  | A1704, Abclonal |
| LIMK1 | (1:1000) |  |  | 3842S, Cell Signaling |
| p-LIMK1 | (1:1000) |  |  | AP0387, Abclonal |
| p38 | (1:1000) |  |  | 8690S, Cell Signaling |
| p-p38 | (1:1000) |  |  | 903501, Biolegend |
| PARP | (1:1000) |  |  | AB32138, Abcam |
| cleaved PARP | (1:1000) |  |  | #9546S, Cell Signaling |
| Tubulin | (1:5000) |  |  | #12004166, BioRad |

**Table S5: Secondary Antibodies**

| **Antibody** | **WB (Dilution)** | **IF (Dilution)** | **Cat.number, company** |
| --- | --- | --- | --- |
| HRP-anti-rabbit IgG | (1:10000) |  | 211-032-171,  Dianova |
| HRP-anti-goat IgG | (1:5000) |  | 705-035-003 Dianova |
| HRP-anti-mouse IgG | (1:10000) |  | 115-035-174, Dianova |
| a-mouse IgG (H+L) - Alexa 555 |  | 1:400 | Mol. Probes |
| α-Mouse IgG (H+L) - Alexa 647 |  | 1:400 | Dianova |
| α-rabbit IgG (H+L) - Alexa 488 |  | 1:400 | Mol. Probes |
| DAPI |  | 1:1000 | Sigma - Aldrich |
| Phalloidin CruzFluor^TM^ 555 Conjugate |  | 1:1000 | ChemCruz |

**Table S6: Cell lines used in this study.**

| **Cell line** | **HeLa (CCl-2 ^TM^)** | **SiHa (HTB-35 ^TM^)** |
| --- | --- | --- |
| **Tissue of origin** | 31 years old, black female with Adenocarcinoma | 55 years old, female, Squamous cell carcinoma |
| **Morphology and growth properties** | epithelial, adherent | epithelial, adherent |
| **Supplier** | ATCC | ATCC |
| **Recommended medium** | MEM (Biowest) | MEM (Biowest) |
| **HPV status** | HPV-18 sequences present | HPV-16 sequences present |
| **pRB status** | positive | positive |
| **p53 status** | Low expression | positive |

**Supplementary Figures:**


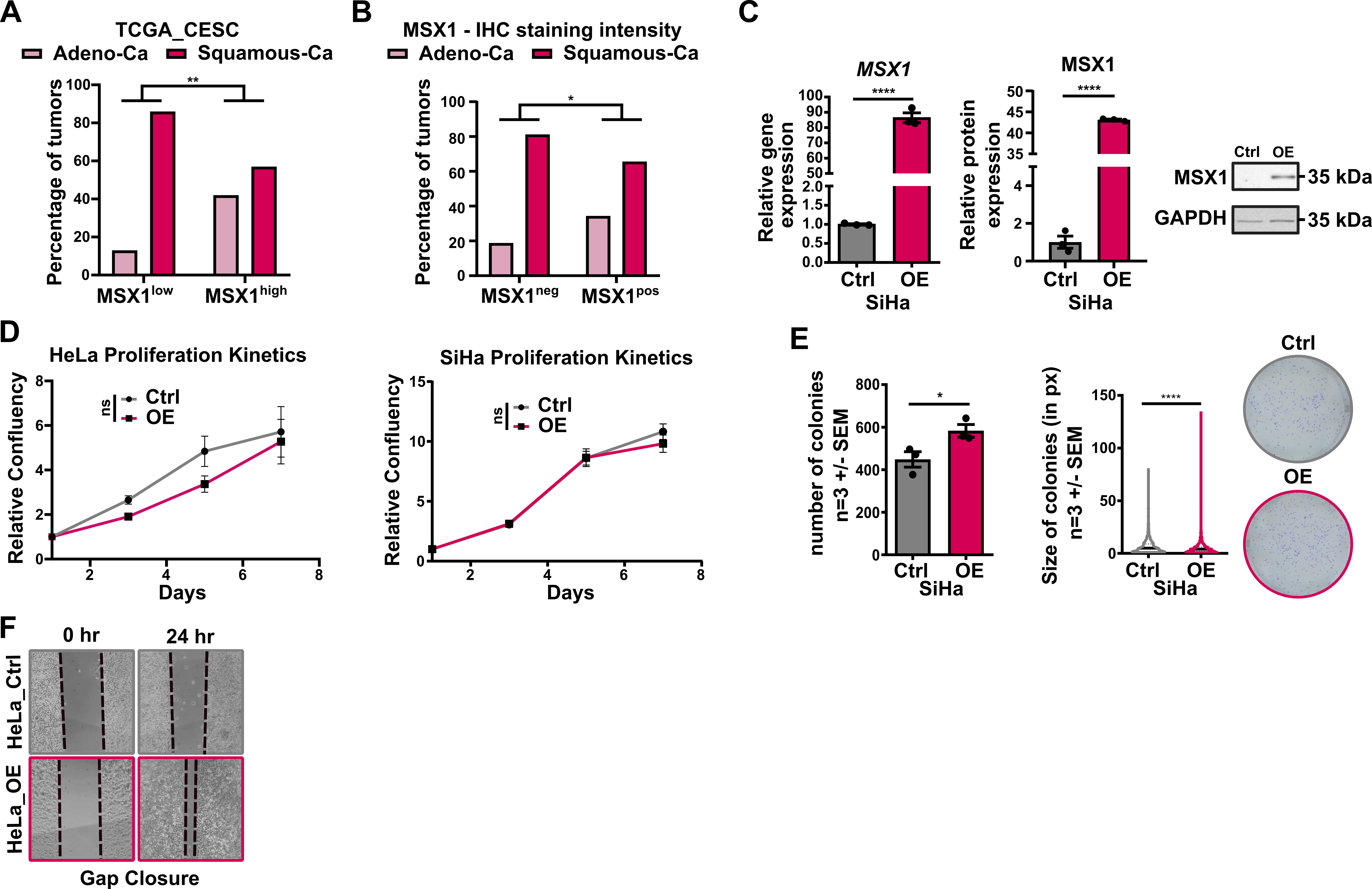


**Figure S1: MSX1 expression leads to a more aggressive phenotype in cervical cancer : A:** Distribution of MSX1-high and MSX1-low tumors in cervical adenocarcinoma (n = 21) and squamous cell carcinoma (n = 280) from the TCGA-CESC dataset. **B:** MSX1 status assessed in an own collective of CC patients (*n=168* squamous cell carcinoma and *n= 45* adenocarcinoma) via immunohistochemisty staining. **C:** Validation of *MSX1*-overexpression (OE) in SiHa cells via RT-qPCR (Left panel) and Western blot (Right panel). **D:** Proliferation assay of HeLa (left panel) and SiHa (right panel) cells overexpressing MSX1 (OE) compared to control cells (Ctrl), quantified using Celigo® imaging. **E:** Colony formation assay of Ctrl and OE SiHa cells. **F:** Representative images of Gap closure assay at 0 hr and 24 hr in HeLa control and MSX1-overexpressing cells. **C-F**: experiments performed in n=3 biological replicates. Statistical test: **A, B**: Chi-Square test; **C, E** (left panel)**:** Student *t*-test; **E** (middle panel): Mann-Whitney test; **D:** AUC follow by Student t-test. *p-val<0.05, **p-val≤0.01, ****p-val≤0.0001.





**Figure S2: High *MSX1* expression favors EMT phenotype in CC cells: A:** RT-qPCR of enriched genes from *HALLMARK_EPITHELIAL_MESENCHYMAL_TRANSITION* (EMT) in SiHa cells control (Ctrl) and MSX1 overexpressing (OE) cells. **B:** Validation of MSX1 KD in stable MSX1 OE HeLa cells compared to control cells (siControl) via Western Blot. **C:** Validation of MSX1 KD and downregulation of EMT genes in stable MSX1 OE HeLa cells via RT-qPCR. **D:** Enrichr pathways enrichment analysis of H3K4me3 regions upregulated upon *MSX1* OE in HeLa cells. **E-F:** IGV tracks depicting occupancy of H3K4me3 and MSX1 at selected EMT (**E**) and RHO/RAC/CDC42 (**F**) gene regions. **G:** Representative images (left) and quantification (right) of phalloidin staining in SiHa control and *MSX1*-OE cells. (Scale Bar: 10 µm). **A-C, G**: Experiments performed in n=3 biological replicates. Statistical test: **A, B, C:** Student t-test, **G:** Mann-Whitney (middle panel) test and Student t-test (right panel). *p-val<0.05, **p-val≤0.01, ***p-val≤0.001, ****p-val≤0.001.

**Figure S3: Interference with RHO signaling by RKI-1447 inhibitor reverts the aggressive phenotype in CC cells: A:** Boyden chamber assay in SiHa Ctrl and *MSX1* OE cells upon treatment with RKI-1447 (10 µM). **B:** Representative images of Gap closure assay at 0 hr and 24 hr in HeLa control and MSX1-overexpressing cells with and without RKI-1447 treatment (10µM). **C:** IF images (left) and quantification (right) of phalloidin staining in SiHa control and *MSX1* OE cells upon 5 µM RKI-1447 treatment. (Scale Bar: 10µm). **D:** Representative images of Gap closure assay at 0 hr and 48 hr in HeLa control and MSX1-overexpressing cells treated with siControl and siFOS. Statistical test: **A:** Student t-test, **C:** Kruskal-Wallis test (upper panel) and One-way ANOVA (lower panel). *p-val<0.05, **p-val≤0.01, ***p-val≤0.001, ****p-val≤0.001.

**Literature**

Prokakis, E., Dyas, A., Grün, R., Fritzsche, S., Bedi, U., Kazerouni, Z. B., Kosinsky, R. L., Johnsen, S. A., & Wegwitz, F. (2021). USP22 promotes HER2-driven mammary carcinoma aggressiveness by suppressing the unfolded protein response. *Oncogene*, *40*(23), 4004–4018. https://doi.org/10.1038/s41388-021-01814-5
