# Supplementary material for: Identification of tumor-promoting functions of the Homeobox family transcription factor MSX1 in cervical cancer
Source: Cell Death Discov. 2026 Jun 5;12:270. doi: 10.1038/s41420-026-03191-y (PMC13241535; doi:10.1038/s41420-026-03191-y)
Supplement: Supplementary file 2 — Original Data [file 41420_2026_3191_MOESM2_ESM.pdf]

Fig. 1E

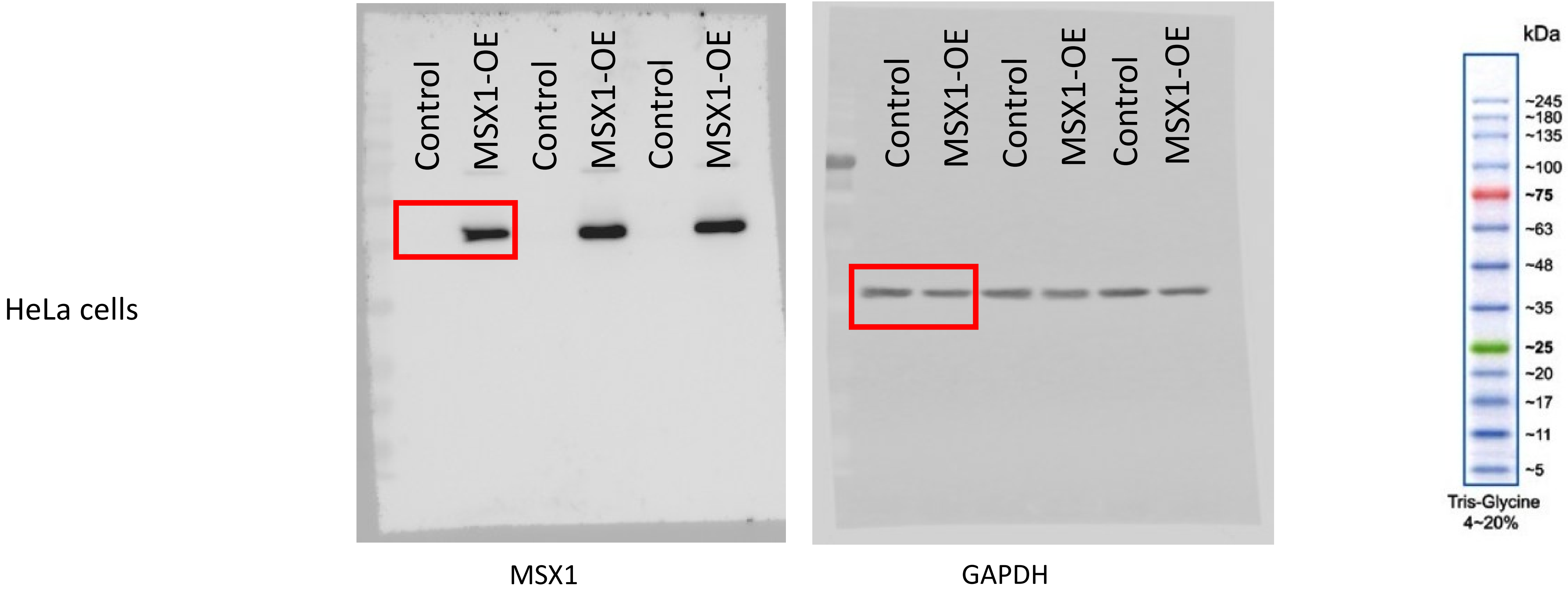
HeLa cells
Control
MSX1-OE
Control
MSX1-OE
Control
MSX1-OE
MSX1
GAPDH
kDa
~245
~180
~135
~100
~75
~63
~48
~35
~25
~20
~17
~11
~5
Tris-Glycine
4~20%

Fig. 2F

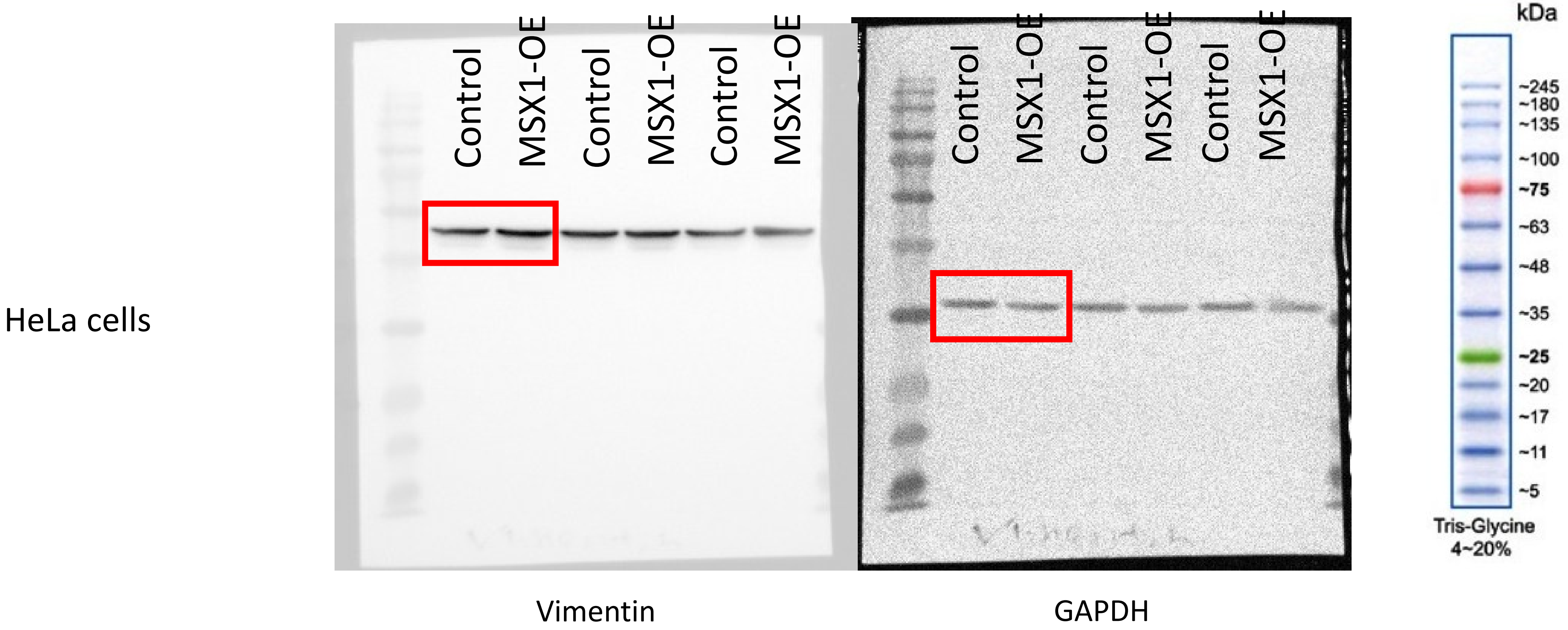
HeLa cells
Control
MSX1-OE
Control
MSX1-OE
Control
MSX1-OE
Control
MSX1-OE
Control
MSX1-OE
Control
MSX1-OE
kDa
~245
~180
~135
~100
~75
~63
~48
~35
~25
~20
~17
~11
~5
Tris-Glycine
4~20%
Vimentin
GAPDH

Fig. 4A

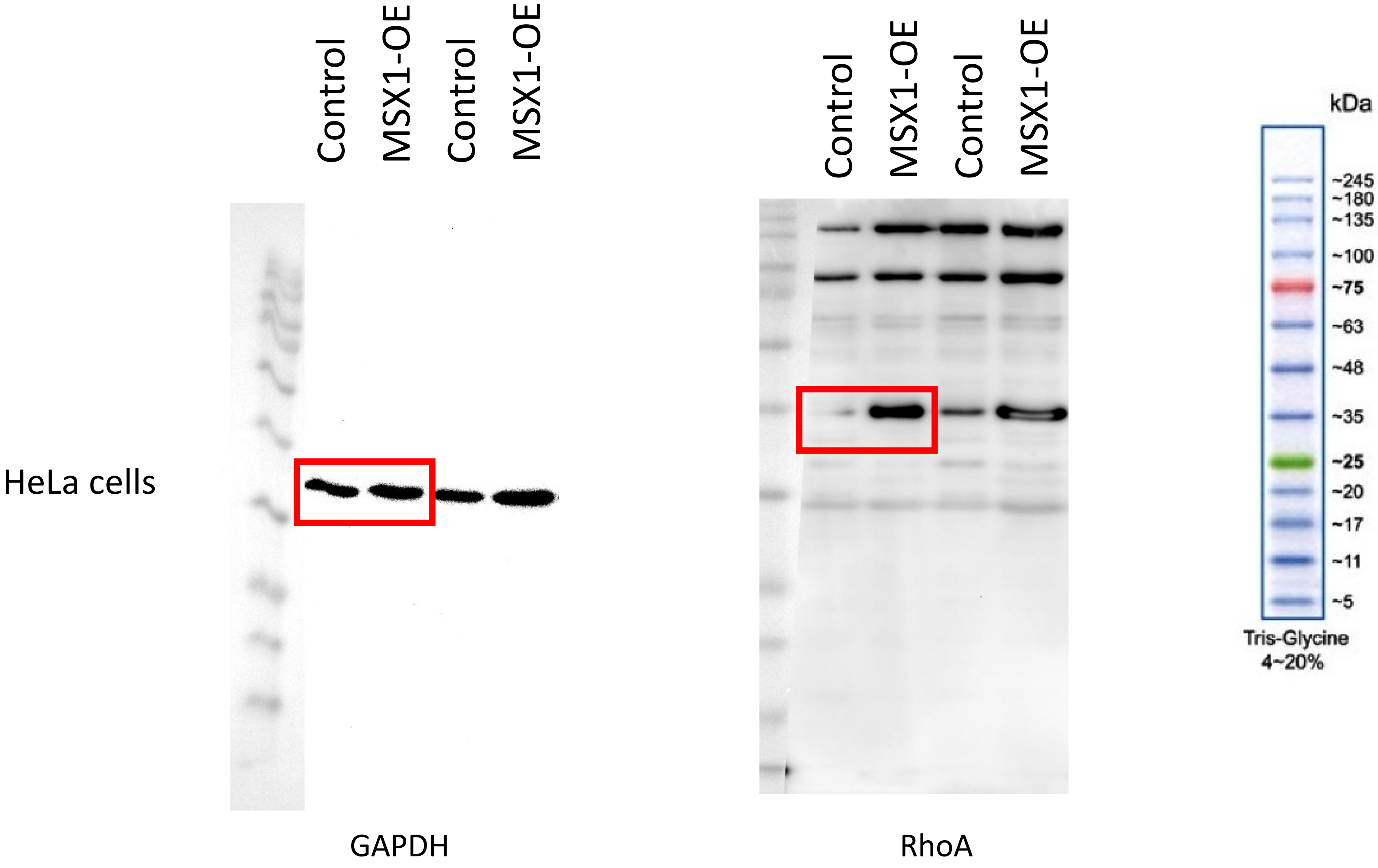
Control
MSX1-OE
Control
MSX1-OE
HeLa cells
GAPDH
Control
MSX1-OE
Control
MSX1-OE
RhoA
kDa
~245
~180
~135
~100
~75
~63
~48
~35
~25
~20
~17
~11
~5
Tris-Glycine
4~20%

Fig. 4B

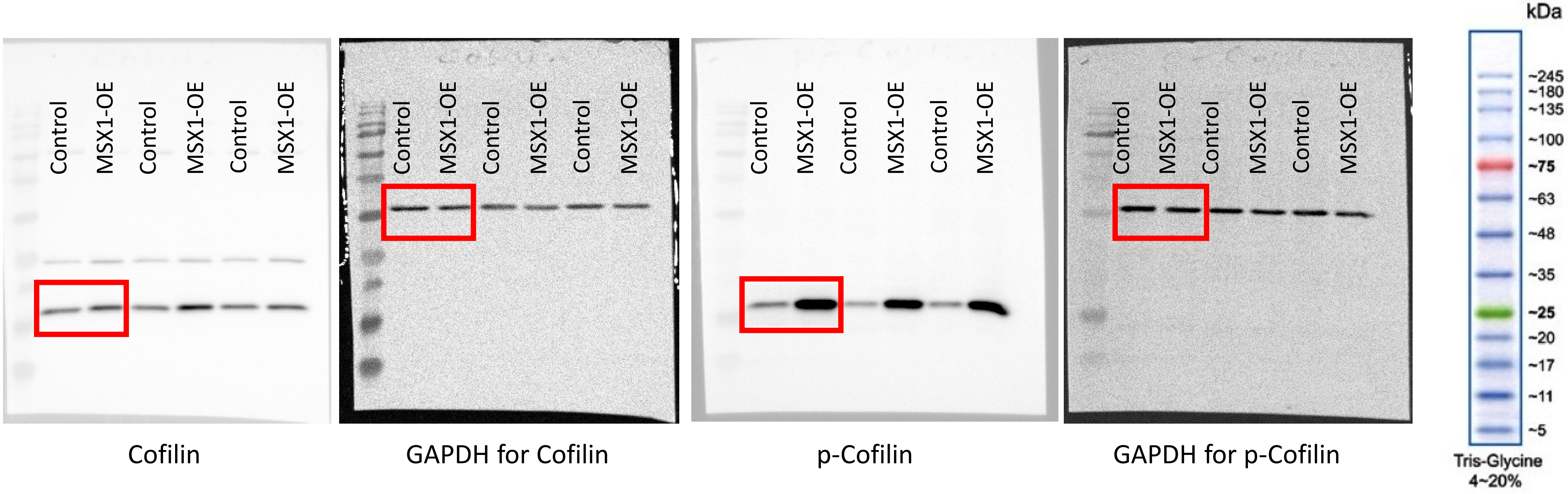

HeLa cells

Fig. 4C

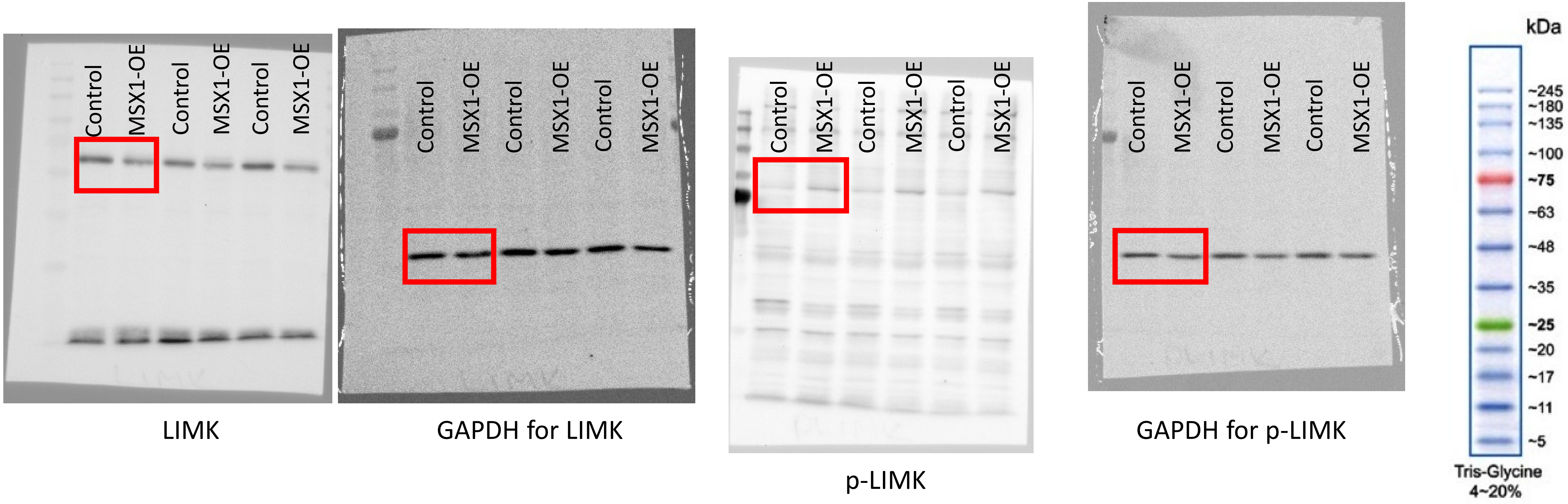

HeLa cells

Fig. 4D

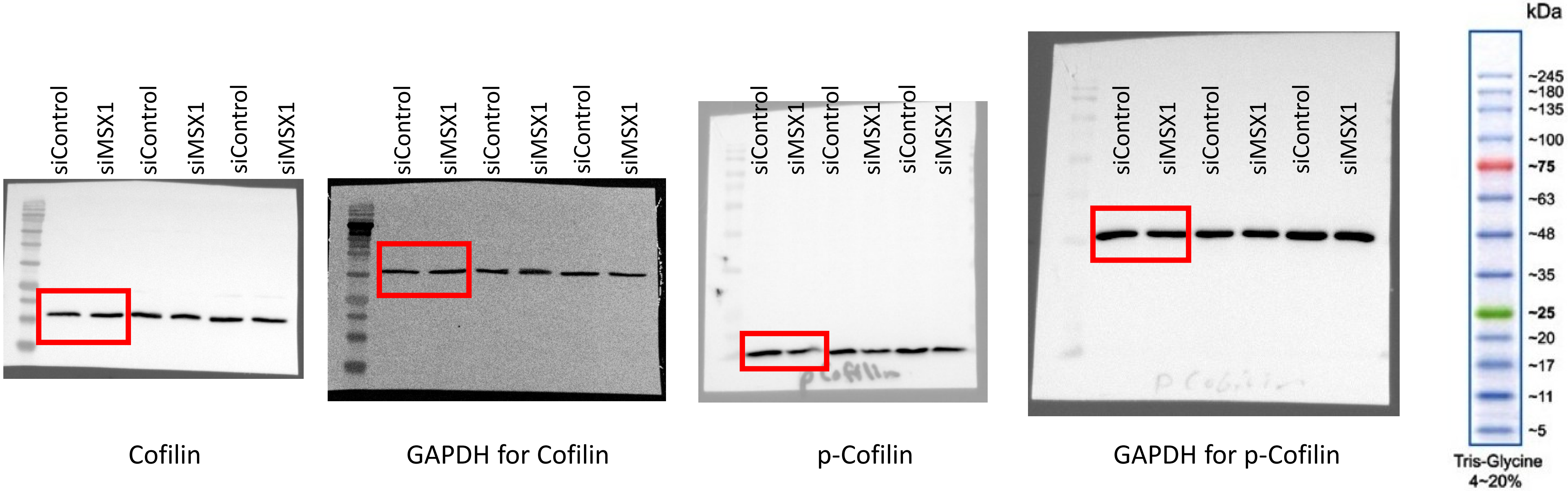

HeLa_OE cells

Fig. 5F

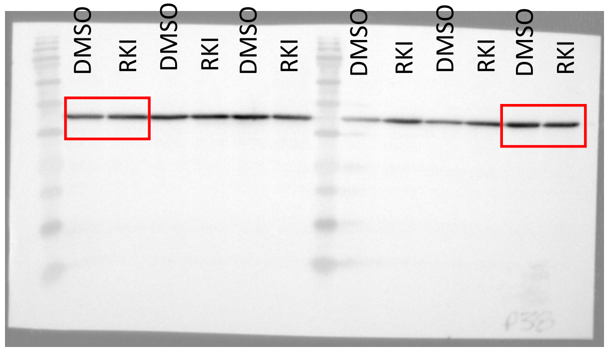

p38

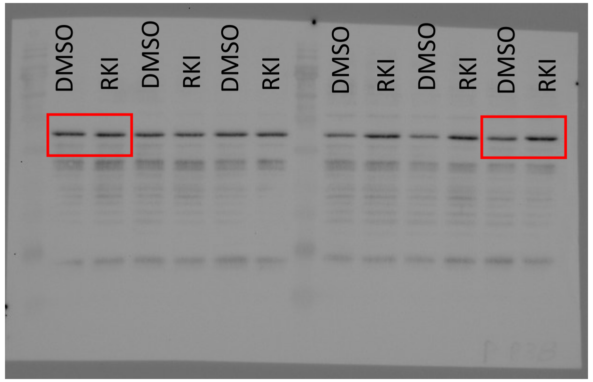

p-p38

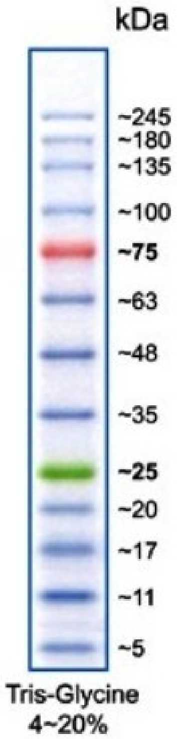

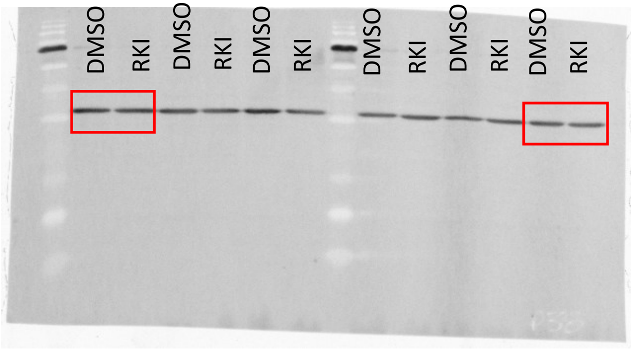

GAPDH for p38

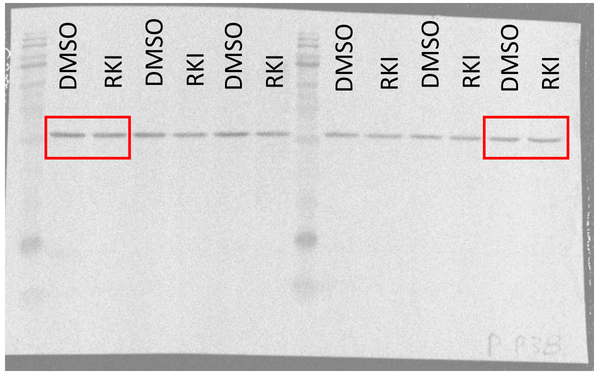

GAPDH for p-p38

HeLa Cells

Fig. 5G

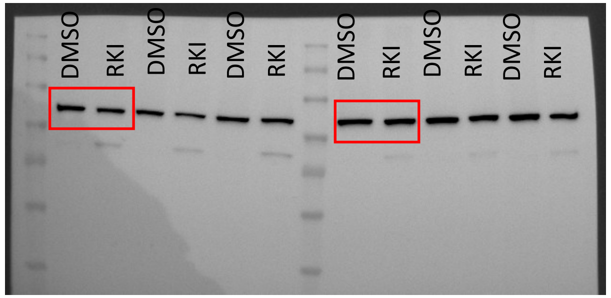

PARP

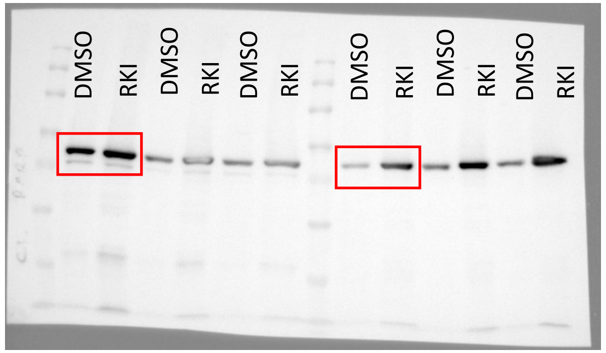

Cl. PARP

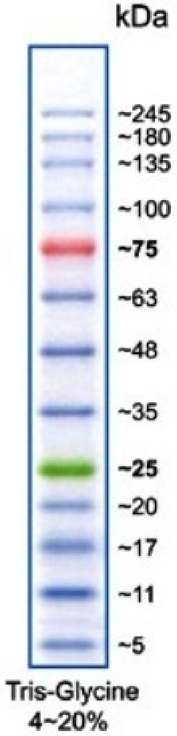

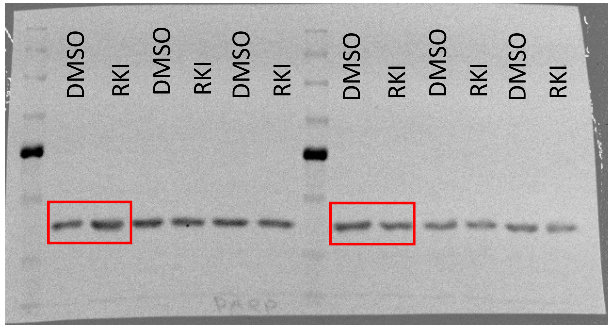

Tubulin for PARP

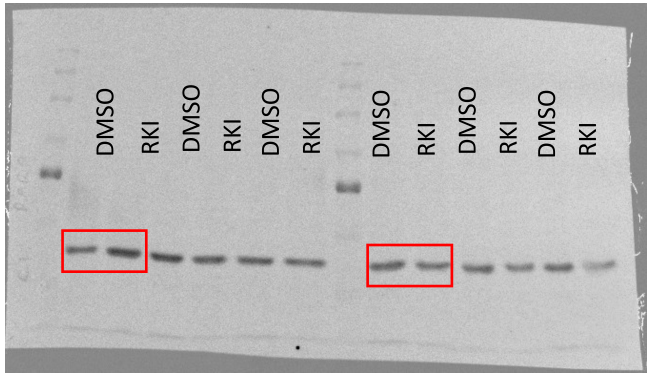

Tubulin for Cl. PARP

HeLa Cells

Fig. S1C

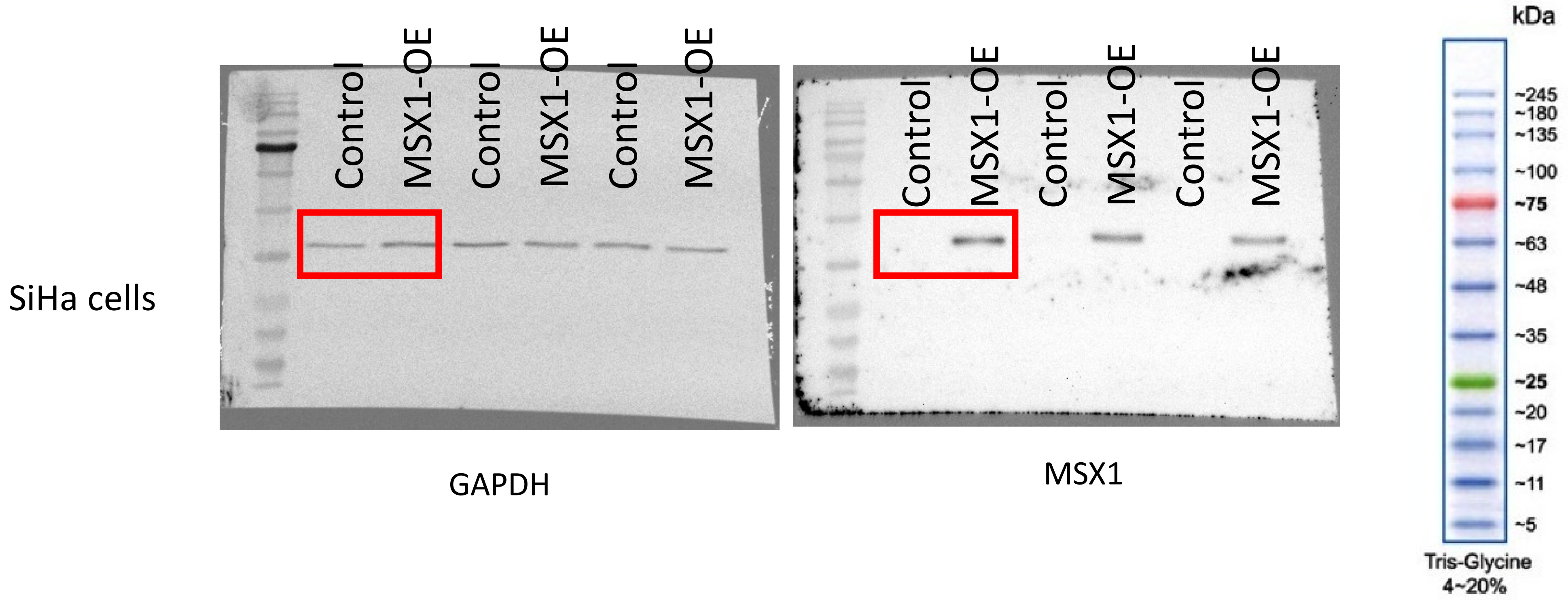
SiHa cells
Control
MSX1-OE
Control
MSX1-OE
Control
MSX1-OE
GAPDH
Control
MSX1-OE
Control
MSX1-OE
Control
MSX1-OE
MSX1
kDa
~245
~180
~135
~100
~75
~63
~48
~35
~25
~20
~17
~11
~5
Tris-Glycine
4~20%
